# Enabling Photoactivated Cross-Linking Mass Spectrometric Analysis of Protein Complexes by Novel MS-Cleavable Cross-Linkers

**DOI:** 10.1016/j.mcpro.2021.100084

**Published:** 2021-04-27

**Authors:** Craig Gutierrez, Leah J. Salituro, Clinton Yu, Xiaorong Wang, Sadie F. DePeter, Scott D. Rychnovsky, Lan Huang

**Affiliations:** 1 Department of Physiology and Biophysics, University of California, Irvine, California, USA; 2 Department of Chemistry, University of California, Irvine, California, USA

**Keywords:** protein-protein interactions, photocross-linking, MS-cleavable cross-linkers, XL-MS, SDASO, DSSO, 26S proteasome, diazirine labeling, NHS-diazirine, protein complexes, 19S RP, 19S regulatory particle, 20S CP, 20S core particle, BMSO, Bismaleimide sulfoxide, a.k.a. 3,3'-sulfinylbis(N-(2-(2,5-dioxo-2,5-dihydro-1H-pyrrol-1-yl)ethyl)propanamide), BSA, bovine serum albumin, CID, collision-induced dissociation, DHSO, Dihydrazide sulfoxide, a.k.a., 3,3’-sulfinyldi(propanehydrazide), DSSO, Disuccinimidyl sulfoxide, LC-MS^n^, liquid chromatography multistage mass spectrometry, MS, mass spectrometry, MS^n^, multistage mass spectrometry, PIP, proteasome-interacting protein, PPI, Protein-protein interaction, SDASO, Succinimidyl diazirine sulfoxide, SDASO-L, Succinimidyl diazirine sulfoxide (long) aka. 2,5-Dioxopyrrolidin-1-yl 3-((2-(3-(3-methyl-3H-diazirin-3-yl)propanamido)ethyl)sulfinyl) propanoate, SDASO-M, Succinimidyl diazirine sulfoxide (medium) aka. 2,5-Dioxopyrrolidin-1-yl 3-((3-(3-methyl-3H-diazirin-3-yl)propyl)sulfinyl)propanoate, SDASO-S, Succinimidyl diazirine sulfoxide (short) aka. 2,5-Dioxopyrrolidin-1-yl 3-((2-(3-methyl-3H-diazirin-3-yl)ethyl)sulfinyl)propanoate, XL-MS, cross-linking mass spectrometry

## Abstract

Cross-linking mass spectrometry (XL-MS) is a powerful tool for studying protein–protein interactions and elucidating architectures of protein complexes. While residue-specific XL-MS studies have been very successful, accessibility of interaction regions nontargetable by specific chemistries remain difficult. Photochemistry has shown great potential in capturing those regions because of nonspecific reactivity, but low yields and high complexities of photocross-linked products have hindered their identification, limiting current studies predominantly to single proteins. Here, we describe the development of three novel MS-cleavable heterobifunctional cross-linkers, namely SDASO (Succinimidyl diazirine sulfoxide), to enable fast and accurate identification of photocross-linked peptides by MS^n^. The MS^n^-based workflow allowed SDASO XL-MS analysis of the yeast 26S proteasome, demonstrating the feasibility of photocross-linking of large protein complexes for the first time. Comparative analyses have revealed that SDASO cross-linking is robust and captures interactions complementary to residue-specific reagents, providing the foundation for future applications of photocross-linking in complex XL-MS studies.

## Highlights

• Development of three mass spectrometry-cleavable photoreactive cross-linkers.
• Characterization of diazirine-based photocross-linking with a standard protein BSA.
• MS^n^-based workflow for photocross-linking analysis of protein complexes.
• SDASO cross-linking mass spectrometry analyses of the yeast 26S proteasome complex.

## Graphical Abstract

**Figure:**
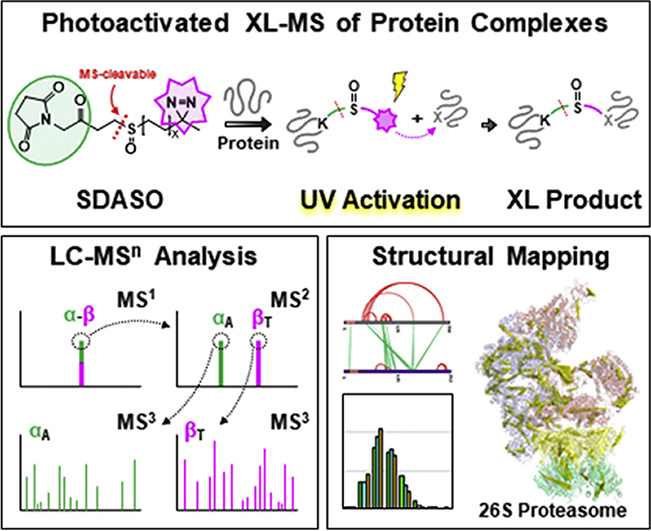

## In Brief

Although photochemistry complements residue-specific chemistry through labeling amino acids nonspecifically, existing photo-cross-linking reagents are thus far inapplicable to multisubunit protein complexes owing to low yields and high complexities of photo-cross-linked products. The development of the three sulfoxide-containing MS-cleavable photoreactive SDASO cross-linkers permits MSn-based analytical workflow for accurate identification of photo-cross-linked peptides, enabling complex PPI profiling for the first time. This work has established a solid foundation for future applications of photo-cross-linking in complex XL-MS studies.

Protein–protein interactions (PPIs) are fundamental to the assembly, structure, and function of protein complexes, which in turn exert control over a diverse array of biological processes integral to cell biology. Cross-linking mass spectrometry (XL-MS) is a unique structural tool capable of studying PPIs because of its ability to simultaneously capture and identify PPIs with interaction contacts from native cellular environments (1, 2, 3, 4, 5). In addition, the residue-specific cross-linkable distances defined by cross-linkers can function as restraints to assist structural modeling and to elucidate architectures of large protein complexes (6, 7, 8). To date, amine-reactive homobifunctional NHS ester cross-linkers have been the most popular reagents in XL-MS studies. This is because of the relatively high occurrence of lysines—particularly at the surfaces of protein structures—as well as the specificity and efficiency of amine-reactive chemistries. Although effective, these reagents alone cannot yield complete PPI maps, as profiling of interaction regions lacking lysines would be difficult. Thus, to complement lysine-reactive reagents, additional amino acid–specific cross-linkers have been developed, including carboxyl-residue (9, 10, 11), sulfhydryl-residue (12, 13), arginine-residue (14), and multiresidue targeting ones (15, 16, 17), clearly expanding PPI coverage. In addition, integration of multiple cross-linkers has improved characterization of PPIs and increased the depth and accuracy of structural analysis (7, 8, 18, 19), demonstrating the benefits of multichemistry-based combinatory XL-MS approaches. However, despite these successes, mapping interaction regions lacking targetable residues by specific chemistry remains challenging.

In recent years, photochemistry has shown great potential in capturing regions inaccessible to residue-specific cross-linkers because of its nonspecific reactivity (2, 3, 20, 21). Various types of photoreactive reagents have been explored in XL-MS studies (13, 22, 23, 24, 25, 26, 27, 28, 29, 30), almost all of which have been heterobifunctional cross-linkers with an amine-reactive specific end and a nonspecific end. Among the commonly used photoreactive groups, alkyl diazirine is most attractive because of its small size, long excitation wavelength, photostability, reactivity, and proven success in XL-MS studies (22, 24, 25, 26, 27, 28, 29, 30). Diazirines are activated by UV light to yield highly reactive carbenes, which then react with an X-H bond (X: C, N, O, S) of any proximal amino acids (24, 25, 27, 29, 30, 31). While promising, the indiscriminate nature of photocross-linking often results in highly complex and low abundance cross-linked products that complicate MS analysis and database searching, thus limiting its application predominantly to single proteins (24, 25, 26, 27, 28, 30). Therefore, to advance photoreactive XL-MS studies for complex PPI mapping, it is essential to develop novel reagents that permit effective MS detection and accurate identification of photocross-linked peptides.

**Fig. 1:**
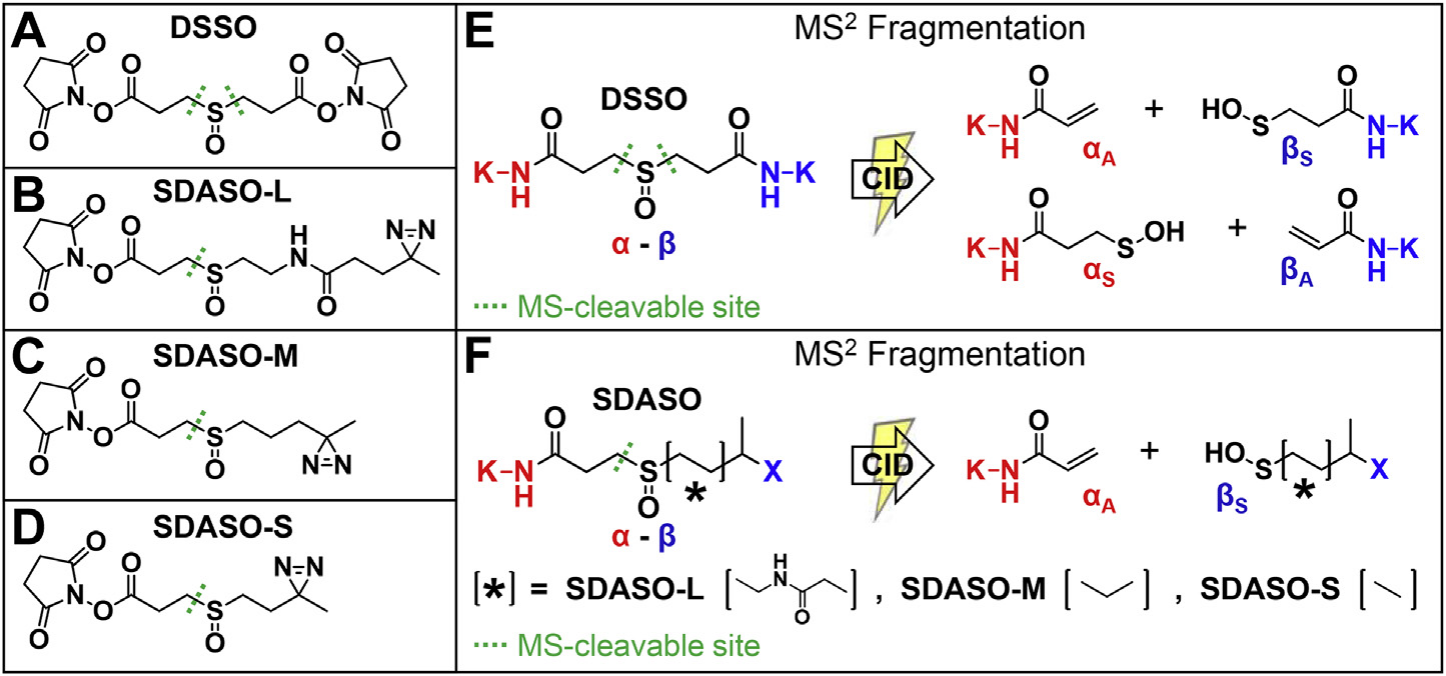
**MS**^**2**^**fragmentation characteristics of sulfoxide-containing MS-cleavable cross-linkers.** Molecular structures of (*A*) DSSO, (*B*) SDASO-L, (*C*) SDASO-M, and (*D*) SDASO-S. *E*, MS^2^ fragmentation of a DSSO interlinked peptide [α-β], representing the characteristics of sulfoxide-containing MS-cleavable cross-linkers with symmetric structures. CID cleavage of either one of the two MS-cleavable C-S bonds physically separates α and β peptide constituents into single peptide chains modified with either alkene (A) (*i.e.*, α_A_, β_A_) or sulfenic acid (S) (*i.e.*, α_S_, β_S_) moieties, the two complementary remnants of the cross-linker after cleavage. *F*, MS^2^ fragmentation of a SDASO interlinked peptide [α-β], signifying the characteristics of sulfoxide-containing MS-cleavable heterobifunctional NHS-diazirine cross-linkers, namely, SDASO-L, -M and -S linkers (*B*–*D*). CID cleavage of the single MS-cleavable C-S bond in SDASO cross-linked peptides produces only one pair of cross-link fragment ions, α_A_/β_S,_ in which α peptide is labeled by NHS ester, and β peptide is labeled by diazirine. CID, collision-induced dissociation; DSSO, disuccinimidyl sulfoxide; MS, mass spectrometry; SDASO-L, Succinimidyl diazirine sulfoxide (long) aka. 2,5-Dioxopyrrolidin-1-yl 3-((2-(3-(3-methyl-3H-diazirin-3-yl)propanamido)ethyl)sulfinyl) propanoate; SDASO-M, succinimidyl diazirine sulfoxide (medium) aka. 2,5-Dioxopyrrolidin-1-yl 3-((3-(3-methyl-3H-diazirin-3-yl)propyl)sulfinyl)propanoate; SDASO-S, succinimidyl diazirine sulfoxide (short) aka. 2,5-Dioxopyrrolidin-1-yl 3-((2-(3-methyl-3H-diazirin-3-yl)ethyl)sulfinyl)propanoate.

MS-cleavable cross-linking reagents have significantly facilitated MS analysis of cross-linked peptides in complex mixtures, because of their unique capability of eliminating the “n-square” problem and permitting effective sequencing of cross-linked peptides (2, 32). To enable robust MS-cleavability, we have previously developed a series of sulfoxide-containing MS-cleavable cross-linking reagents (*e.g.*, disuccinimidyl sulfoxide [DSSO]) (Fig. 1*A*) (10, 12, 33, 34, 35, 36). The MS-labile C-S bonds adjacent to the sulfoxide can be preferentially fragmented before peptide backbone cleavage upon collision-induced dissociation (CID), physically separating the two cross-linked peptide constituents for individual sequencing. Notably, this predictable fragmentation occurs independent of cross-linking chemistry, peptide charge, and peptide sequence. These unique characteristics allow straightforward and unambiguous identification of cross-linked peptides by MS^n^ analysis coupled with conventional database searching tools. Sulfoxide-containing MS-cleavable cross-linkers have been successfully applied to not only study PPIs *in vitro* (33, 37, 38, 39) and *in vivo* (34, 39) but also to dissect structural dynamics of protein complexes (8, 40, 41). Thus, to expedite the identification of photocross-linked peptides, we have developed three sulfoxide-containing MS-cleavable heterobifunctional NHS-diazirine cross-linkers with varied lengths, namely, SDASO (Succinimidyl diazirine sulfoxide)-L (long), -M (medium) and -S (short). These SDASO reagents represent the first class of sulfoxide-containing MS-cleavable heterobifunctional photoreactive cross-linkers. To illustrate their capabilities, we have characterized SDASO cross-linkers with a standard protein bovine serum albumin (BSA) and applied them to map PPIs of affinity purified yeast 26S proteasome. Our results demonstrate that MS-cleavability enables accurate identification of photocross-linked peptides and that the SDASO-based XL-MS workflow is well-suited for probing PPIs in complex samples. In addition, comparison with residue-specific XL-MS data has determined that SDASO cross-linking is robust and captures PPIs complementary to existing reagents.

## Experimental Procedures

### Synthesis and Characterization of SDASO Cross-Linkers

Three SDASO cross-linkers were designed, synthesized, and analyzed in this work (Fig. 1), including SDASO-L, SDASO-M, and SDASO-S. Their synthesis and characterization are described in supplemental Fig. S1 and supplemental Methods.

### Purification of the Yeast 26S Proteasome Complexes

RPN11-TAP strain was used for yeast proteasome purification as previously described (42). Briefly, the yeast strain was cultured in YEPD medium (1% yeast extract, 2% peptone, and 2% dextrose) at 30 °C until stationary phase, then the cells were collected and washed with ice-cold water. The yeast cells were loaded into 20 ml syringe and pushed into liquid nitrogen to get yeast frozen “noodles” which were ground in a Cryomill into frozen powder. The yeast frozen powder was resuspended in a lysis buffer containing 50 mM sodium phosphate (pH 8.0), 5 mM magnesium chloride, 1 mM ATP, 1× protease inhibitor (Sigma), then sonicated at 15 W with 30 s on and 30 s off for three cycles, and pellet was spun down at 15,000 rpm for 15 min. The supernatant was bound to IgG resin (MP Biomedical#55961) for 2 h at 4 °C with rotation. The IgG resin was washed with 50 bed volume of wash buffer (50 mM sodium phosphate [pH 7.4], 100 mM NaCl, 5 mM magnesium chloride, 1 mM ATP), then 20 bed volume of TEV cleavage buffer (50 mM Sodium phosphate [pH 7.4], 10% glycerol, 1 mM EDTA, 1 mM DTT and 1 mM ATP), and the bound proteasome was cleaved overnight with TEV at 4 °C. The resulting proteasome was concentrated with 30K cutoff Centricon devices (EMD Millipore UFC903024).

### XL-MS Analysis of BSA and 26S Proteasome

Protein cross-linking was performed similarly to previous studies with some modifications (12, 25). Briefly, for SDASO cross-linking of BSA, 50 μl of 50 μM protein solution in PBS buffer (pH 7.4) was reacted in triplicate with SDASO-L, SDASO-M, or SDASO-S in molar ratio of 1:50, respectively, for 1 h at 25 °C in the dark. The NHS reactive ends were quenched with the addition of ammonium bicarbonate at a 50-fold excess for 10 min at 25 °C in the dark. Then NHS ester labeled proteins were transferred into Millipore Microcon Ultracel PL-30 (30-kDa filters) and washed three times with 300 μl PBS buffer. Diazirine cross-linking was activated by UV irradiation, which was carried out on ice ~5 cm from the light source in an UV light chamber (Analytikjena UVP Cross-linker CL-1000L) and irradiated at 365 nm for 30 min.

The affinity purified yeast 26S proteasome was (supplemental Methods) cross-linked by SDASO linkers similarly as described above. To determine the optimal SDASO cross-linking conditions, we have performed initial XL-MS experiments of the yeast 26S proteasome using 5, 10, 20, and 40 mM SDASO, respectively. As a result, 20 mM SDASO yielded the highest number of cross-link identifications and was determined as the optimal cross-linking condition for this work. Specifically, 100 μg of the 26S proteasome in PBS buffer (pH 7.4) was cross-linked in triplicate with 20 mM SDASO-L, SDASO-M, and SDASO-S, respectively. In addition, 100 μg of the yeast 26S proteasome in PBS buffer (pH 7.4) was cross-linked with 2.5 mM or 5 mM DSSO for 1 h at 25 °C temp similarly as described (8), and the reactions were quenched with the addition of ammonium bicarbonate at a 50-fold excess for 10 min. Then cross-linked proteins were transferred into Millipore Microcon Ultracel PL-30 (30-kDa filters) for digestion.

### Digestion of Cross-Linked Proteins

The resulting cross-linked products were subjected to enzymatic digestion using a FASP protocol (43). Briefly, cross-linked proteins on FASP filters were reduced/alkylated and digested with Lys-C/trypsin or chymotrypsin as described (8, 33). The resulting digests were desalted, and cross-linked peptides were enriched by size-exclusion chromatography before LC MS^n^ analysis (10, 44).

### Experimental Design and Statistical Rationale

Three SDASO cross-linkers were designed and characterized in this work with a standard protein BSA and an affinity purified yeast proteasome. Each SDASO XL-MS experiment was performed in biological triplicate under optimized conditions. To evaluate the effect of enzymatic digestion on SDASO results, chrymotrypsin digestion was performed for SDASO-L cross-linked 26S proteasome in biological triplicate as well. In total, nine SDASO XL-MS experiments for BSA analysis and 12 SDASO XL-MS experiments for the yeast 26S proteasome. Cross-validation was carried out among the results obtained from the three SDASO linkers. To further evaluate the SDASO results, DSSO XL-MS experiments of the yeast 26S proteasome were performed in two biological replicates. Reproducibility in XL-MS experiments was assessed at the level of cross-linked peptide sequences and sites, respectively.

### LC-MS^n^ Analysis and Identification of Cross-Linked Peptides

Cross-linked peptides were analyzed by LC-MS^n^ using a Thermo Scientific Dionex UltiMate 3000 system online coupled with an Orbitrap Fusion Lumos mass spectrometer (8). A 50 cm × 75 μm Acclaim PepMap C18 column was used to separate peptides over a gradient of 1% to 25% ACN in 106 min for BSA and in 166 min for the 26S proteasome at a flow rate of 300 nl/min. MS^1^ scans (375–1500 m/z, resolution at 120,000) were performed with the AGC target set to 4e5 in top speed mode with a cycle time of 5 s. For MS^n^ analysis, 3+ and up charged ions were selected for MS^2^-CID in FT mode, followed by top four data-dependent MS^3^ acquisition method (45). A targeted MS^3^ acquisition was also used for DSSO cross-linked peptides by utilizing the mass difference between alkene- and thiol-modified ion pairs (31.9721 Da) (45). For MS^2^ scans, the resolution was set to 30,000, the AGC target 5e4, the precursor isolation width was 1.6 m/z, and the maximum injection time was 100 ms for CID. The CID-MS^2^ normalized collision energy was 25%. For MS^3^ scans, CID was used with a collision energy of 35%, the AGC target was set to 2e4, and the maximum injection time was set to 120 ms.

### Identification of Cross-Linked Peptides

MS^n^ data were extracted using MSConvert (ProteoWizard 3.0.10738) and analyzed similarly as previously described (8). Briefly, the extracted MS^3^ data were subjected to a developmental version of Protein Prospector (v.6.0.0) for database searching, using Batch-Tag against a custom random concatenated database (a total of 988 entries) derived from BSA and 493 *Saccharomyces cerevisiae* protein sequences that were identified from the affinity purified yeast 26S proteasomes. The mass tolerances for parent ions and fragment ions set as ±20 ppm and 0.6 Da, respectively. Trypsin or chymotrypsin was set as the enzyme with three or four maximum missed cleavages allowed, respectively. A maximum of four variable modifications were allowed, including cysteine carbamidomethylation, protein N-terminal acetylation, methionine oxidation, and N-terminal conversion of glutamine to pyroglutamic acid. In addition, three defined modifications representing alkene on uncleaved lysines, thiol and sulfenic fragment moieties on any amino acid (AAs) were selected for each respective SDASO cross-linker. Specifically, for SDASO-L cross-links: alkene (C_3_H_2_O; +54 Da), sulfenic acid (C_7_H_13_NO_2_S; +175 Da), and thiol (C_7_H_11_NOS; +157 Da). For SDASO-M cross-links: alkene (C_3_H_2_O; +54 Da), sulfenic acid (C_5_H_10_OS; +118 Da), and thiol (C_5_H_8_S; +100 Da). For SDASO-S cross-links: alkene (C_3_H_2_O; +54 Da), sulfenic acid (C_4_H_8_OS; +104 Da), and thiol (C_4_H_6_S; +86 Da). For DSSO cross-links, three defined modifications on uncleaved lysines are: alkene (C_3_H_2_O; +54 Da), sulfenic acid (C_3_H_4_O_2_S; +104 Da), and thiol (C_3_H_2_SO; +86 Da) (33). Owing to the conversion of the SDASO sulfenic acid moiety to the thiol moiety alongside backbone fragmentation during MS^3^ analysis, we have incorporated such neutral loss in Batch-tag to facilitate the identification of sulfenic acid-modified peptides during database searching using Protein Prospector. The in-house program xl-Tools was used to validate and summarize cross-linked peptides based on MS^n^ data and database searching (33, 39). To ensure the confidence in cross-link identification, we examined whether peptide sequences with ambiguous diazirine labeling sites have been identified repeatedly and found that the majority of those were verified by redundant identifications of same peptide sequences but different site localizations. Owing to the labeling capability of diazirine, we cannot exclude the possibility of the ambiguous sites being targeted. Further manual inspection was performed to examine peptide identification and site localization. Following integration of MS^n^ data, there were no decoy hits found in the final lists of identified cross-linked peptides for all XL-MS experiments except for the tryptic digests of SDASO-L cross-linked 26S proteasome with a FDR ≤0.08%. To ensure the reliability of the identified cross-links, cross-validation was performed among the three biological replicates for each linker and across the three SDASO reagents reported here.

### Analysis of the Identified Cross-Links

Circular 2-D XL-maps were constructed using the CX-Cirus online application (http://cx-circos.cloudapp.net/), and linear 2-D XL-maps were created using the online application xiNET Crosslink Viewer (http://crosslinkviewer.org). 3-D maps were generated based on BSA (PDB: 4F5S), 26S proteasome structures (PDB:4CR2 (s1), 4CR3 (s2), 4CR4 (s3), and 5MPD (s4)). The state-specific cross-links of the 26S proteasome were determined by mapping them onto s1-s4 state models, which are summarized in supplemental Table S3*A*.

### Analysis of Amino Acid Preference for Diazirine Labeling

The unique K-X linkages identified for both BSA (supplemental Table S1*B*) and 26S (supplemental Table S2*B*) were used to assess diazirine labeling frequency at specific amino acids, in which only the peptide constituents labeled by diazirine were used for evaluation. The weighted occurrence values of diazirine-labeled AAs were determined based on their localization precision similarly as described (31). Briefly, for a given cross-linked peptide identified with *n* possible ambiguous sites, the weighted score *Wx* of each site *x* is determined as *ax,r*, which is the preference of reagent *r* toward residue at site *x*. So, *Wx* = *ax,r/n*. Assuming the preference for any AA in a given peptide is equal to 1, then *Wx = 1/n*. For all cross-linked peptides identified from the three biological replicates for each SDASO linker, the total weighted score for a given site *x* was calculated as $Wx^{\prime }=\overset{m}{\underset{i=1}{\sum }}Wx$, in which *m* is the total number of *x* in the identified cross-linked peptides. Then, the likelihood of carbene insertion at any site *x* was calculated as: $Px=Wx^{\prime }/\overset{k}{\underset{i=1}{\sum }}Wi^{\prime }$ (sum of all weighted scores for every x sites).

### Distribution of Random Cross-Links

XWalk (46) was utilized to generate random cross-link distribution. Alpha carbon distances from lysine residues to all other residues (X) were generated individually using Euclidean distances only, skipping solvent-path-distance calculations. The maximum distance was set to 100 Å for BSA and 300 Å for 26S proteasome to capture all possible residue linkage combinations in each protein/protein complex. Individual data for all residue combinations were compiled to generate histograms corresponding to random distributions for BSA, 26S, 20S, and 19S, respectively.

## Results

### Designs of MS-Cleavable NHS-Diazirine Heterobifunctional Cross-Linkers

To advance photoreactive cross-linkers for complex PPI mapping, we sought to create novel sulfoxide-containing MS-cleavable NHS-diazirine heterobifunctional cross-linking reagents to cross-link lysines to any nearby AAs. It is noted that all of our previous sulfoxide-containing MS-cleavable cross-linkers are homobifunctional and carry two symmetric MS-cleavable C-S bonds adjacent to the central sulfoxide (Fig. 1, *A* and *E*) (10, 12, 33, 34, 35). Owing to the structural differences in reactive groups and their targeted residues, this symmetry is not retained in heterobifunctional cross-linkers. Recently, we have explored effects of spacer arm structures on MS-cleavability of sulfoxide-containing cross-linkers and identified an asymmetric spacer arm structure (47) that maintains the characteristic and predictable fragmentation expected of symmetric sulfoxide-containing MS-cleavable cross-linkers (10, 12, 33, 34, 35, 36). This unique asymmetric spacer arm region carries a sulfoxide group that divides the spacer arm into two halves, *i.e.*, a fixed half identical to DSSO with the sulfoxide and carbonyl group separated by ‘3’ bond lengths, and a flexible half. Based on this design, we constructed three MS-cleavable heterobifunctional SDASO cross-linkers composed of a fixed NHS ester end and a flexible diazirine side with varying lengths from the center sulfoxide (*i.e.*, long, 12.5 Å; medium, 10.2 Å; short, 7.7 Å), well within the distance range suited for studying PPIs (2) (Fig. 1, *B*–*D*). The synthesis routes and chemical analyses of SDASOs were detailed here (supplemental Fig. S1 and supplemental Methods).

### Fragmentation Characteristics of SDASO Cross-Linked Peptides

**Fig. 2:**
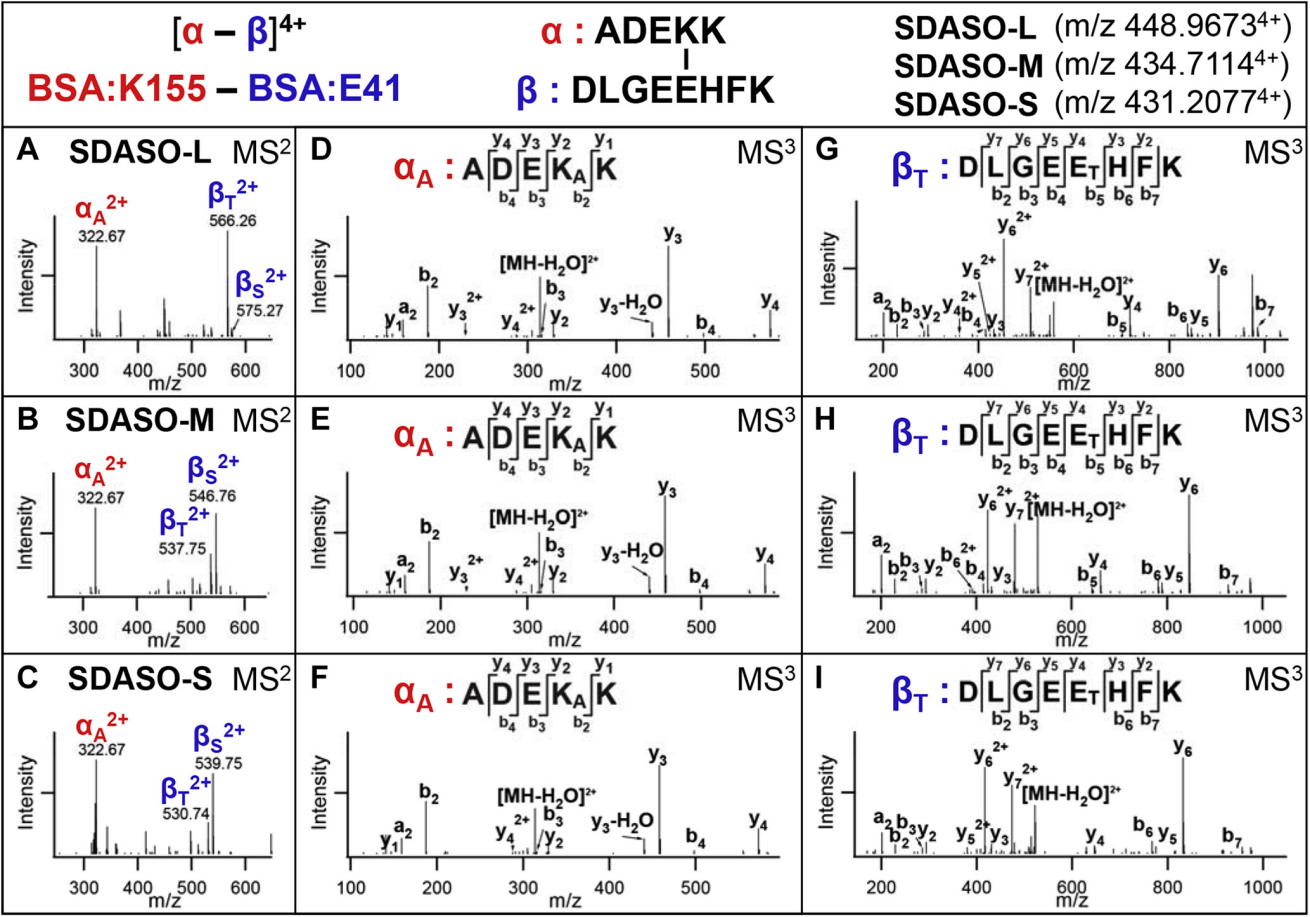
**MS**^**n**^**analyses of representative SDASO-L, SDASO-M and SDASO-S interlinked peptides of BSA.** MS^1^ analyses determined the parent masses of the same peptides (α-β) cross-linked by SDASO-L (m/z 448.9673^4+^), SDASO-M (m/z 434.7114^4+^), SDASO-S (m/z 431.2077^4+^), respectively. MS^2^ spectra of the (*A*) SDASO-L, (*B*) SDASO-M, and (*C*) SDASO-S cross-linked peptides. MS^3^ spectra of the SDASO-L MS^2^ fragment ions: (*D*) α_A_ (m/z 322.67^2+^) and (*E*) β_T_ (m/z 556.26^2+^), the SDASO-M MS^2^ fragment ions: (*F*) α_A_ (m/z 322.67^2+^) and (*G*) β_T_ (m/z 537.75^2+^), and the SDASO-S MS^2^ fragment ions: (*H*) α_A_ (m/z 322.67^2+^) and (*I*) β_T_ (m/z 530.74^2+^). The selected BSA cross-linked peptide was identified as ^152^ADEKK^156^ interlinked to ^37^DLGEEHFK^44^ by MS^3^ analyses (*A*–*I*), in which the K155-E41 linkage was determined. MS^n^, multistage mass spectrometry; SDASO-L, Succinimidyl diazirine sulfoxide (long) aka. 2,5-Dioxopyrrolidin-1-yl 3-((2-(3-(3-methyl-3H-diazirin-3-yl)propanamido)ethyl)sulfinyl) propanoate; SDASO-M, succinimidyl diazirine sulfoxide (medium) aka. 2,5-Dioxopyrrolidin-1-yl 3-((3-(3-methyl-3H-diazirin-3-yl)propyl)sulfinyl)propanoate; SDASO-S, succinimidyl diazirine sulfoxide (short) aka. 2,5-Dioxopyrrolidin-1-yl 3-((2-(3-methyl-3H-diazirin-3-yl)ethyl)sulfinyl)propanoate.

Based on our recent studies on asymmetric sulfoxide-containing cross-linkers (47), only the C-S bond at the NHS ester end in SDASO should be preferentially cleaved during CID. Thus, a single pair of MS^2^ fragment ions is expected for all three SDASO cross-linkers (Fig. 1*F*). For an SDASO interlinked peptide (α-β), cleavage during CID physically separates the two cross-linked constituents and thus leads to the detection of two characteristic fragment ions (α_A_/β_S_) carrying remnants of SDASO. The α_A_ fragment contains a cross-linked lysine modified with the alkene (A) moiety, whereas the β_S_ fragment contains a photocross-linked amino acid modified with a sulfenic acid (S) moiety. Because the NHS ester side of all three SDASO reagents are identical to half of DSSO, the expected alkene moieties are the same as seen in DSSO cross-linked peptides (Fig. 1*E*). In contrast, the three SDASO cross-linkers yield three different sulfenic acid moieties because of spacer arm differences in the diazirine end (Fig. 1*F*). As previously noted for other sulfoxide-containing cross-linkers (10, 12, 33, 34, 35, 47), the sulfenic acid moiety typically undergoes dehydration to become a more stable and dominant unsaturated thiol (T) moiety, leading to the detection of β_T_ (supplemental Fig. S2*A*). To examine whether SDASO cross-linked peptides produce the expected fragmentation, standard protein BSA was cross-linked by the three SDASO cross-linkers separately, and the resulting peptide digests were analyzed by LC MS^n^. As illustrated (Fig. 2), each MS^n^ analysis of the same BSA peptides interlinked by the three SDASO reagents yielded a dominant MS^2^ fragment pair (α_A_/β_T_) as predicted. These resultant MS^2^ fragment ions representing single peptide chains were then subjected to individual MS^3^ analyses, permitting unambiguous identification of both cross-linked peptide sequences and cross-linking sites. As a result, the respective cross-links between BSA:K155 and BSA:E41 were identified for all SDASO linkers.

Similar to residue-specific cross-linkers, SDASO cross-linking can also result in dead-end and intralinked peptides. For SDASO cross-linkers, two types of dead-end peptides are expected as both reactive ends can be hydrolyzed (supplemental Fig. S2, *B* and *C*). For NHS ester dead-ends, the resulting fragment ions would carry thiol moieties (supplemental Fig. S2*B*), whereas the MS^2^ fragment ion of diazirine dead-end peptides would be labeled with an alkene moiety (supplemental Fig. S2*C*). These predicted MS^2^ fragmentations were demonstrated by respective SDASO dead-end peptides of BSA (supplemental Fig. S3). Similarly, for SDASO intralinked peptides, a single fragment would be expected, containing both an alkene and thiol modification (supplemental Fig. S2*D*). Exemplary MS^n^ spectra of the three SDASO intralinked peptides of BSA further demonstrated the anticipated fragmentation (supplemental Fig. S4).

Collectively, the three types of SDASO cross-linked peptides fragment as predicted during CID to generate characteristic and predictable MS^2^ products, which enable their simplified and accurate identification by MS^n^ analysis in the same way as other sulfoxide-containing cross-linked peptides (10, 12, 33, 34, 35, 47).

### SDASO XL-MS Analysis of BSA

**Fig. 3:**
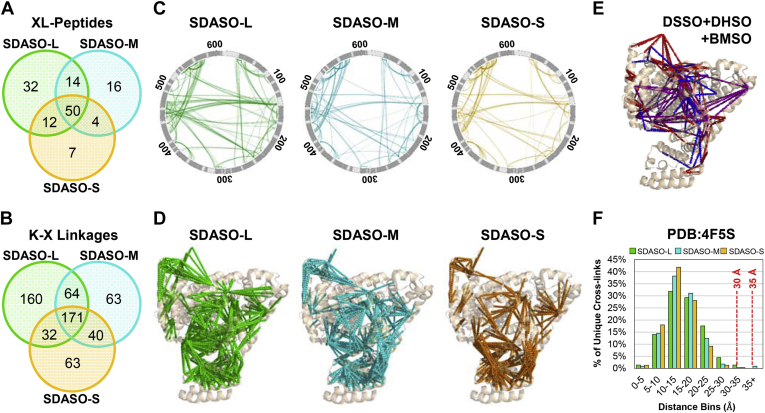
**Comparisons of BSA cross-link data by the three SDASO linkers.** Comparisons of (*A*) cross-linked peptide sequences and (*B*) residue-to-residue linkages of BSA obtained from SDASO-L, SDASO-M, and SDASO-S XL-MS experiments. *C*, circular 2-D SDASO XL-maps of BSA based on SDASO-L, -M and -S cross-links respectively. Helical secondary structures are designated by dark gray regions. *D*, 3-D SDASO XL-maps of BSA on its crystal structure (PDB: 4F5S) based on SDASO-L, -M, and -S cross-links, respectively. *E*, 3-D XL-map of BSA (PDB: 4F5S) generated based on the combined cross-links from DSSO (*blue*)+DHSO (*red*)+BMSO (*purple*) XL-MS experiments (10, 12). *F*, distance distribution plots of the identified SDASO cross-links to the BSA structure (PDB: 4F5S) (SDASO-L: ≤35 Å, SDASO-M and -S: ≤30 Å). Note: Colors schemes represent specific linkers: SDASO-L: *light green*, SDASO-M: *light blue*, and SDASO-S: *gold orange*. SDASO-L, Succinimidyl diazirine sulfoxide (long) aka. 2,5-Dioxopyrrolidin-1-yl 3-((2-(3-(3-methyl-3H-diazirin-3-yl)propanamido)ethyl)sulfinyl) propanoate; SDASO-M, succinimidyl diazirine sulfoxide (medium) aka. 2,5-Dioxopyrrolidin-1-yl 3-((3-(3-methyl-3H-diazirin-3-yl)propyl)sulfinyl)propanoate; SDASO-S, succinimidyl diazirine sulfoxide (short) aka. 2,5-Dioxopyrrolidin-1-yl 3-((2-(3-methyl-3H-diazirin-3-yl)ethyl)sulfinyl)propanoate; XL-MS: cross-linking mass spectrometry.

To evaluate the performance of the three SDASO cross-linkers, we first carried out XL-MS analyses of BSA with three biological replicates each. Based on the general workflow (supplemental Fig. S5), LC MS^n^ analyses resulted in a total of 556 unique SDASO-L, 405 SDASO-M, 324 SDASO-S interlinked BSA peptides, encompassing 427, 338, 306 unique K-X linkages, respectively (supplemental Table S1, *A* and *B*). Here, X represents any of the 20 common AAs. Although the three SDASO cross-linkers produced similar amounts of XL-MS data, SDASO-S consistently generated the least number of cross-linked peptides. This is not entirely surprising as short linkers are more stringent on distance constraints between two cross-linkable residues, as seen in residue-specific linkers (19, 48). Because of the nonspecificity, it is suspected that photoactivated reaction would lead to increased variance in cross-linked products compared with residue-specific cross-linkers. To test this, we first compared the sequences of identified SDASO cross-linked peptides of BSA for each linker without considering their site localization. Interestingly, all three linkers displayed similar reproducibility with considerably high overlaps (~64%) among their corresponding three biological replicates (supplemental Fig. S6, *A*–*C*). When examining residue-to-residue (*i.e.*, K-X) linkages, all three linkers also exhibited good reproducibility, with overlaps of 50% for SDASO-L, 42% for SDASO-M, and 43% for SDASO-S among their three respective biological replicates (supplemental Fig. S6, *D*–*F*). Intriguingly, the observed residue-to-residue reproducibility of the three SDASO linkers is also quite comparable with cross-linkers with specific chemistries (*i.e.*, DSSO, dihydrazide sulfoxide, a.k.a., 3,3’-sulfinyldi(propanehydrazide) [DHSO], and bismaleimide sulfoxide, a.k.a. 3,3'-sulfinylbis(N-(2-(2,5-dioxo-2,5-dihydro-1H-pyrrol-1-yl)ethyl)propanamide) [BMSO]) (10, 12), indicating the robustness and reliability of SDASO cross-linking. When comparing among the three SDASO linkers, 37% of cross-linked peptide sequences and 29% of their corresponding K-X linkages of BSA were found in common (Fig. 3, *A* and *B*). Our results indicate that the three SDASO linkers have similar efficiency in cross-linking BSA and mapped a considerable number of shared regions but also yielded unique cross-linked peptides and sites.

### Evaluation of SDASO Cross-Links of BSA

To explore the interaction coverage of BSA by SDASO cross-linking, we derived both 2-D and 3-D XL-maps based on the identified K-X linkages (Fig. 3, *C* and *D*). In comparison with our published XL-MS data of BSA using DSSO (amine-reactive), DHSO (acidic residue–reactive), and BMSO (cysteine-reactive) cross-linkers (supplemental Fig. S7, *A*–*D*, Fig. 3*E*) (10, 12), SDASO XL-MS resulted in the highest number of cross-linked peptides and contact sites, thus enabling the generation of the most extensive interaction coverages. As shown, interactions within the central core of BSA are broadly mapped by all types of linkers, while interactions at the N and C termini of BSA are best profiled by the SDASO linkers (Fig. 3, *C*–*E*, supplemental Fig. S7, *A*–*D*). These results demonstrate that SDASO cross-linking is effective for mapping interactions of single proteins and generates structural information complementary to residue-specific cross-linkers.

Among the 20 common AAs that can be targeted by diazirine, arginine has the longest side-chain. Considering the spacer arm lengths of SDASOs (*i.e.*, SDASO-L [12.5 Å], SDASO-M [10.2 Å], SDASO-S [7.7 Å]), side-chain lengths of lysine (6.3 Å) and arginine (7.1 Å), as well as backbone flexibility and structural dynamics, the theoretical upper limits for the Cα-Cα distances of SDASO cross-links between a lysine (NHS reactive end) and any AA (diazirine reactive end) would be ≤35 Å for SDASO-L and ≤30 Å for SDASO-M and SDASO-S. To validate the SDASO cross-links of BSA, we mapped all of the identified cross-links onto the crystal structure of BSA (PDB: 4F5S) (Fig. 3*F*). As a result, 100% of SDASO-L, 99% of SDASO-M, and 100% of SDASO-S linkages were satisfied with Cα-Cα distances well below their respective maximum thresholds (Fig. 3*F*, supplemental Table S1*C*), supporting the validity of the SDASO cross-links. Notably, the average distances of SDASO cross-links also corresponded well with the linker lengths: 15.8 ± 5.8 Å (SDASO-L), 15.1 ± 5.8 Å (SDASO-M), and 14.1 ± 4.8 Å (SDASO-S). Although the spacer arm lengths are comparable, SDASO cross-links displayed higher satisfaction rates and lower average distances than those of DSSO and DHSO cross-links of BSA (10). This may be due to the fact that amino acids other than arginine would result in distances less than the expected upper limits (29). To further validate, we compared distance distributions of SDASO data with that of random cross-links in BSA (supplemental Fig. S8, *A*–*C*), which displayed statistically significant differences, demonstrating that SDASO cross-links do not represent purely random cross-links.

### SDASO-Based XL-MS Analysis of the Yeast 26S Proteasome Complex

**Fig. 4:**
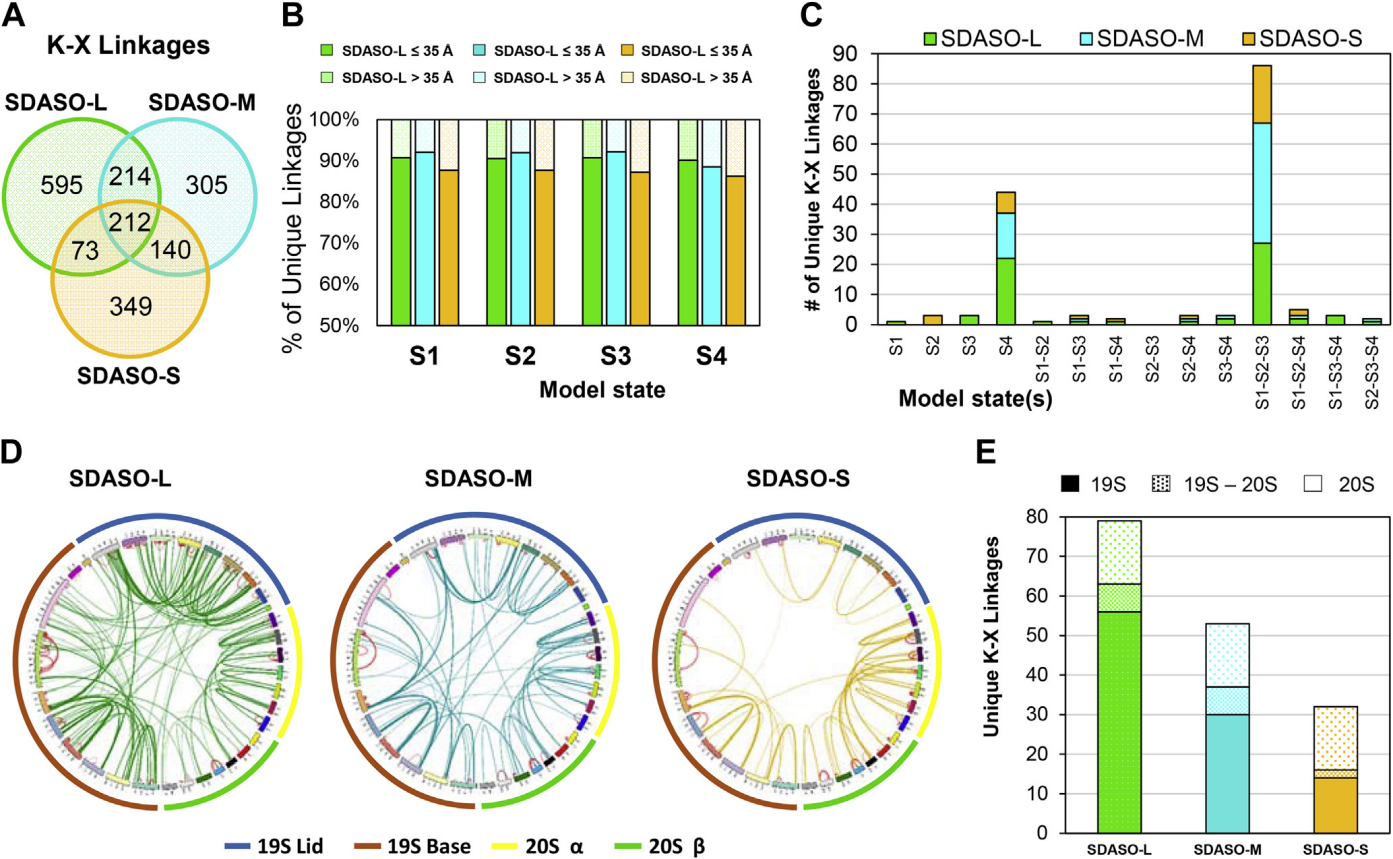
**SDASO XL-MS data summary of the yeast 26S proteasome.***A*, comparisons of cross-linked peptide sequences and residue-to-residue linkages of the 26S proteasome obtained from SDASO-L, SDASO-M, and SDASO-S XL-MS experiments. *B*, respective distance satisfaction rates of SDASO -L, -M, and -S cross-links (SDASO-L: ≤35 Å, SDASO-M and -S: ≤30 Å) mapped onto the four model states of the yeast 26S (PDB: 4CR2 (s1), 4CR3 (s2), 4CR4 (s3), and 5MPD (s4)). *C*, distribution of unique state-specific K-X linkages of SDASO-L, -M, and -S across the 14 possible combinations of one, two, or three out of the four states. *D*, circular 2-D XL-maps of the yeast 26S Proteasome for SDASO-L, -M, -S linkers. Intrasubunit linkages are shown *red* and intersubunit linkages are illustrated based on the linkers: *light green* (SDASO-L), *light blue* (SDASO-M), or *gold orange* (SDASO-S). *Outer circle* represents the subunits within the two subcomplexes of the 26S proteasome, *i.e.*, the 19S RP (Lid [*blue*] and base [*dark orange*]) and 20S CP (α ring [*yellow*] and β ring [*green*]). *E*, distributions of SDASO-L, -M, and -S cross-links corresponding to three categories of intersubunit interactions: 20S-20S (*light shade* [*top*]), 19S-20S (*dotted shade* [*middle*]), and 19S-19S (*solid shade* [*bottom*]). Note: linker-specific color schemes: SDASO-L: *light green*, SDASO-M: *light blue*, and SDASO-S: *gold orange*. Proteasome subunits are color coded as described (supplemental Table S3*C*). SDASO-L, Succinimidyl diazirine sulfoxide (long) aka. 2,5-Dioxopyrrolidin-1-yl 3-((2-(3-(3-methyl-3H-diazirin-3-yl)propanamido)ethyl)sulfinyl) propanoate; SDASO-M, succinimidyl diazirine sulfoxide (medium) aka. 2,5-Dioxopyrrolidin-1-yl 3-((3-(3-methyl-3H-diazirin-3-yl)propyl)sulfinyl)propanoate; SDASO-S, succinimidyl diazirine sulfoxide (short) aka. 2,5-Dioxopyrrolidin-1-yl 3-((2-(3-methyl-3H-diazirin-3-yl)ethyl)sulfinyl)propanoate; XL-MS: cross-linking mass spectrometry.

To access the feasibility of photoactivated cross-linking for complex PPI mapping, we performed SDASO XL-MS analyses of affinity purified yeast 26S proteasome complex. This 33-subunit protein degradation machine consists of two subcomplexes, the 19S regulatory particle (RP) and 20S core particle (CP) (49). The 19S RP contains 19 subunits that are assembled into the lid (*i.e.*, Rpn3, Rpn5-9, Rpn11, Rpn12, Rpn15/Sem1) and base (Rpt1-6, Rpn1-2, Rpn10, Rpn13) subcomplexes, whereas the 20S CP is composed of 14 subunits (α1-7, β1-7) that form four stacked 7-member ring structures in the order of αββα. With three biological replicates for each linker, LC MS^n^ analyses of tryptic digests of SDASO cross-linked complexes resulted in the identification of 1165 SDASO-L, 1133 SDASO-M, and 902 SDASO-S unique cross-linked peptides within the 26S proteasome (supplemental Table S2*A*), representing 1094 SDASO-L (496 intersubunit and 598 intrasubunit), 871 SDASO-M (416 intersubunit and 455 intrasubunit), and 777 SDASO-S (255 intersubunit and 522 intrasubunit) unique K-X linkages (supplemental Table S3*A*). As a result, 43% of SDASO-L, 52% of SDASO-M, and 60% of SDASO-S cross-linked peptide sequences (supplemental Fig. S9, *A*–*C*), as well as 29% of SDASO-L, 37% of SDASO-M, and 38% of SDASO-S K-X linkages were found reproducible among their respective biological replicates (supplemental Fig. S9, *D*–*F*), comparable to BSA data. These results further support the robustness of SDASO cross-linking. When comparing XL-MS data among the three linkers, we found that the number of SDASO cross-links of proteasomes increased with spacer arm lengths of the linkers, similar to BSA data. However, the resulting cross-link data among the three linkers shared considerably fewer in common for proteasomes than for BSA, with overlaps of 16% *versus* 37% for cross-linked peptide sequences and of 11% *versus* 29% for K-X linkages (Figs. 3, *A* and *B* and 4*A*, supplemental Fig. S10).These results suggest that spacer arm lengths of SDASO linkers play a more significant role in capturing interactions within protein complexes, most likely attributed to the presence of both interprotein and intraprotein interactions. Thus, the use of the three SDASO linkers is beneficial not only for result cross-validation but also for comprehensive PPI mapping of protein complexes.

As additional enzymatic digestions are known to increase sequence coverage in XL-MS analyses using residue-specific cross-linkers (44), we expected that similar results would be obtained for SDASO linkers. To test this, we performed chymotrypsin digestion of SDASO-L cross-linked proteasomes with three biological replicates. LC MS^n^ analyses of chymotryptic digests resulted in the identification of a total of 776 unique SDASO-L cross-linked peptides of the 26S proteasome (supplemental Table S2, *A* and *B*), representing 804 SDASO-L unique K-X linkages, comparable to the trypsin XL-MS data as described above (supplemental Table S3*A*). While the reproducibility of XL-MS data was somewhat similar for both chymotryptic and tryptic digests of SDASO-L cross-linked proteasomes (supplemental Figs. S8, *A* and *D* and S11, *A* and *B*), their overlaps of cross-linked peptide sequences and K-X linkages were quite limited (~10%) (supplemental Fig. S11, *C* and *D*). This confirms that additional enzymatic digestion could facilitate the expansion of PPI coverages. Thus, tryptic and chymotryptic datasets of SDASO-L were combined, yielding a total of 1711 unique SDASO-L K-X linkages for subsequent analyses (supplemental Table S3*A*).

### Validation of Proteasome Cross-Links by Structural Mapping

It is known that the 26S proteasome is a dynamic entity and possesses multiple conformational states to fulfill its function (49, 50). To validate SDASO cross-links, we mapped the identified K-X linkages onto the four known structures of the yeast 26S proteasome that represent its progression through an ATP-driven functional cycle: s1 (PDB:4CR2), s2 (PDB:4CR3), s3 (PDB:4CR4) and s4 (PDB:5MPC) (51, 52). As a result, the average distance satisfaction rates of the identified K-X linkages across the four models for each linker were found to be very similar: 91% for SDASO-L (≤35 Å), 91% for SDASO-M (≤30 Å), 87% for SDASO-S (≤30 Å), with an overall variation less than 1% (Fig. 4*B*, supplemental Fig. S12, *A*–*D*). To eliminate the possibility of the identified SDASO cross-links being random, we have compared their distance distributions with that of random cross-links of the 26S proteasome (supplemental Fig. S13, *A*–*C*). As shown, SDASO distributions are significantly different from the random distribution, similar to a previous report on the yeast 26S proteasome using residue-specific cross-linkers (53), further demonstrating the reliability of our identified cross-links.

Additionally, we noticed a group of SDASO linkages that appeared to fit better with a subset of models (supplemental Table S3*A*), suggesting the presence of conformational heterogeneity in the sample. To examine this, we classified a total of 159 SDASO cross-links as structural state-specific, because they were satisfied only by one, two or three out of the four models. We then grouped these differentially satisfied cross-links into 14 state-specific combinations to infer the presence of preferred structural states. As illustrated in Figure 4*C*, among all combinations, two major categories were detected for the three SDASO linkers, representing 82% of the total state-specific SDASO cross-links. One of them contained cross-links (54%) satisfied only by s1-s3 states but not by the s4 state, implying the presence of s1, s2 and/or s3 states in the purified proteasome. The other described cross-links (~28%) satisfied only by the s4 state, indicating presence of that state. These two groups of state-specific cross-links represent 28 protein interactions, half of which describe connectivity within the 20S CP. The remaining half embody interactions within the 19S, particularly concerning Rpn11 and Rpn1. The results correlate well with the fact that these regions are expected to undergo significant conformational changes during state conversions of the 26S proteasome (51, 52).

When considering intersubunit and intrasubunit cross-links separately, the latter has a slightly higher distance satisfaction when mapped to known structures (intrasubunit: SDASO-L: 98%, SDASO-M: 96%, and SDASO-S: 88% *versus* intersubunit: SDASO-L: 79%, SDASO-M: 86%, and SDASO-S: 86%) (supplemental Fig. S14, *A*–*M*). This is expected as intersubunit interactions are typically more dynamic. Coincidentally, the majority of nonsatisfied intersubunit linkages also localized to the 19S RP (supplemental Fig. S15, *A*–*M*), which is known to have diverse conformations (50). Collectively, structural mapping supports the validity of the identified SDASO cross-links and suggests the existence of multiple states in our purified proteasome.

### Comparison of SDASO XL-Maps of the 26S Proteasome

To further evaluate the performance of SDASO in complex PPI mapping, we generated 2-D XL-maps of the 26S proteasome based on unique K-X linkages identified by each SDASO linker (Fig. 4*D*). A total of 135 nonredundant PPIs (103 intersubunit and 32 intrasubunit) within the 26S proteasome were determined based on 2427 K-X linkages identified by the three SDASO linkers, including 119 from SDASO-L (79 intersubunit and 30 intrasubunit), 81 from SDASO-M (53 intersubunit and 28 intrasubunit), and 61 from SDASO-S (32 intersubunit and 29 intrasubunit) (supplemental Table S3*C*). While ~20% of intersubunit interactions were identified across all three linkers (21/103), each linker contributed unique interactions (SDASO-L 42/103, SDASO-M 16/103, and SDASO-S 5/103). The intersubunit interactions of the 26S proteasome captured by each linker can be classified into three categories based on proteasome subcomplexes: 19S-19S (56 SDASO-L, 30 SDASO-M, and 14 SDASO-S), 19S-20S (7 SDASO-L, 7 SDASO-M, and 2 SDASO-S), and 20S-20S (16 each for SDASO-L, -M, and -S), as illustrated in Figure 4*E*. The differences in the PPIs captured by SDASO linkers are most likely related to their spacer arm lengths. Nevertheless, these results indicate that SDASO cross-linking covers a diverse range of protein interactions and that each SDASO linker contributes to mapping the comprehensive interaction network within the 26S proteasome.

### DSSO XL-MS Analysis of the 26S Proteasome

To better assess SDASO cross-link data, we performed a set of XL-MS experiments on the yeast 26S proteasome using DSSO for comparison. LC MS^n^ analyses identified a total of 2254 unique DSSO cross-linked peptides of proteasomes from two biological replicates, representing 1115 K-K linkages (659 intersubunit and 456 intrasubunit) and describing 107 intersubunit and 30 intrasubunit interactions (supplemental Tables S2*C* and S3*C*). While the overlap (65%) of DSSO cross-linked peptide sequences between the two biological replicates was comparable to those of SDASO data (57%~70%) (supplemental Fig. S16*A*), the reproducibility of DSSO residue-to-residue (*i.e.*, K-K) linkages was higher (~65%) (supplemental Fig. S16*B*) than those of SDASO data (29%~38%). The increased variation in identified SDASO cross-link sites is expected as nonspecific cross-linking chemistry is inherently more variable. Nonetheless, these comparisons further demonstrate that SDASO cross-linking is robust on targetable interaction regions.

Next, we mapped DSSO cross-links onto the four conformational states (s1-s4) of the yeast 26S proteasome (51, 52) and determined that on average ~75% of DSSO K-K linkages were satisfied (≤30 Å) across all four models (supplemental Fig. S17, *A* and *B*). Interestingly, a total of 114 DSSO cross-links were also found to be state-specific cross-links, as described above. However, the distribution of cross-links across the 14 state-specific combinations was somewhat different from SDASO data (supplemental Figs. S4*C* and S17*C*). In addition to the notable representations of s4 (30%) and s1-s3 states (15%) as seen in SDASO data, respective state-specific DSSO cross-links satisfied only by s1 state (~9%), s3 state (~8%), and s2-s3-s4 states (~14%) were markedly detected. These DSSO state-specific cross-links further support the presence of multiple conformational states of the 26S proteasome. Similar to SDASO data, intrasubunit DSSO cross-links were much better satisfied than intersubunit linkages for all four models (intra: 89% *versus* inter: 62%) (supplemental Fig. S17, *D*, *F*–*I*), and most of the violating DSSO intersubunit cross-links were attributed to the 19S RP (supplemental Fig. S17, *E*, *J*–*M*). Taken together, DSSO XL-MS data corroborate well with SDASO results, confirming the structural heterogeneity of affinity purified 26S proteasome and the dynamic nature of the 19S RP.

### Comparison of SDASO and DSSO Cross-Linking of Proteasomes

**Fig. 5:**
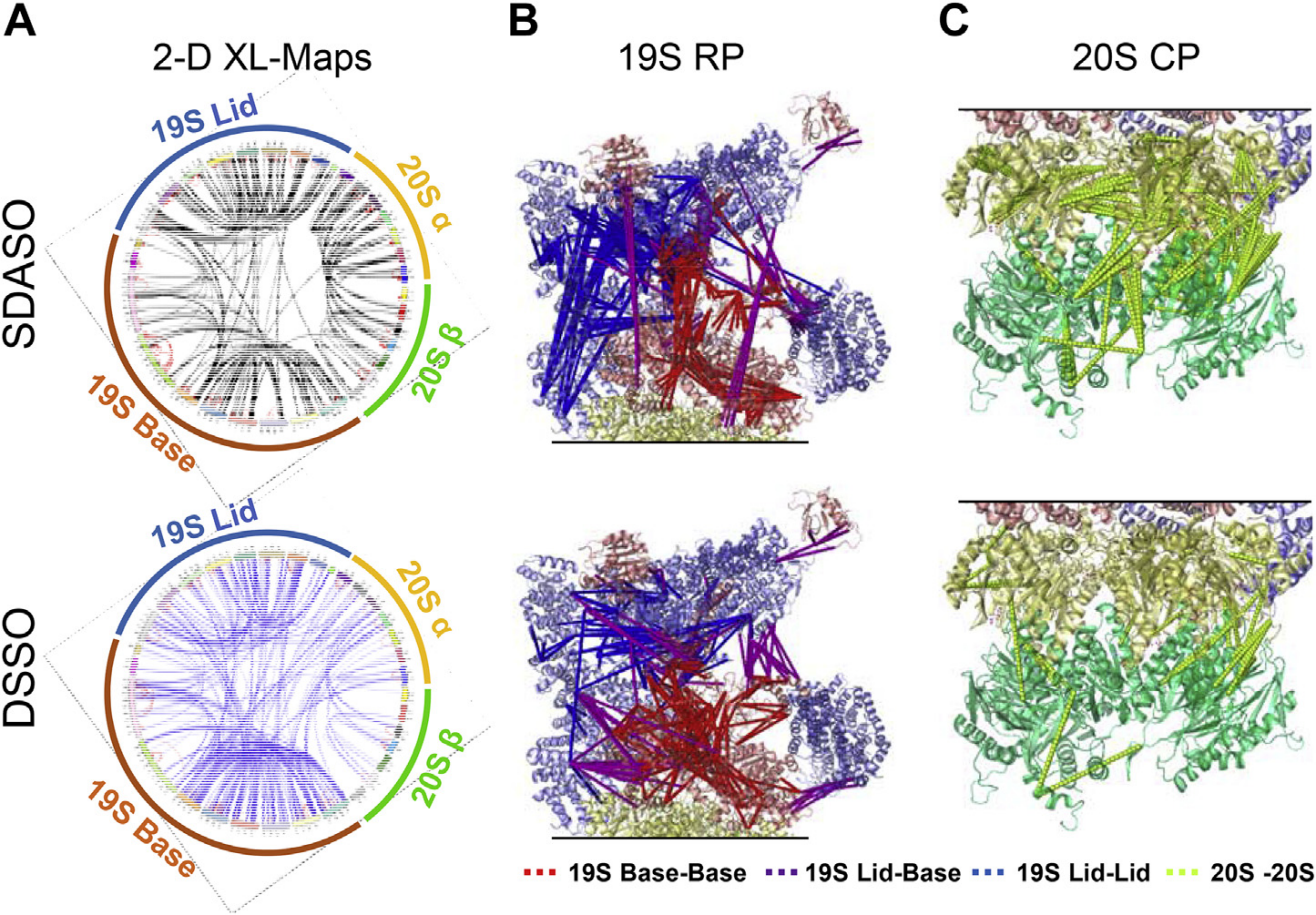
**SDASO and DSSO XL-maps of the yeast 26S Proteasome.***A*, circular 2-D XL-maps of the 26S Proteasome derived from all three SDASO linkers (*top*) and DSSO (*bottom*). Intrasubunit linkages are shown *red* and intersubunit linkages are colored *black* for SDASO and *blue* for DSSO. *Outer circle* represents the subunits within the two subcomplexes of the 26S proteasome, *i.e.*, the 19S RP (Lid [*blue*] and base [*dark orange*]) and 20S CP (α ring [*yellow*] and β ring [*green*]). *B*, 3D XL-maps of the 19S RP using SDASO (*top*) and DSSO (*bottom*) cross-links, in which 19S lid subunits are colored *light blue* and base subunits colored *light red*. Cross-links are also color coded: 19S lid-lid (*blue lines*), 19S base-base (*red lines*), 19S lid-base (*purple*). *C*, 3D XL-maps of the 20S CP based on SDASO (*top*) and DSSO (*bottom*) cross-links, in which 20S α subunits are colored as *light yellow*, 20S β subunits as *aqua green*, and 20S linkages as *lime green*. Note: high-resolution structure of the yeast 26S proteasome (PDB: 4CR2 (s1) was used for the maps in (*B*) and (*C*). Subunits are color coordinated as shown in supplemental Table S3*C*. CP, core particle; DSSO, disuccinimidyl sulfoxide; RP, regulatory particle; SDASO, succinimidyl diazirine sulfoxide.

**Fig. 6:**
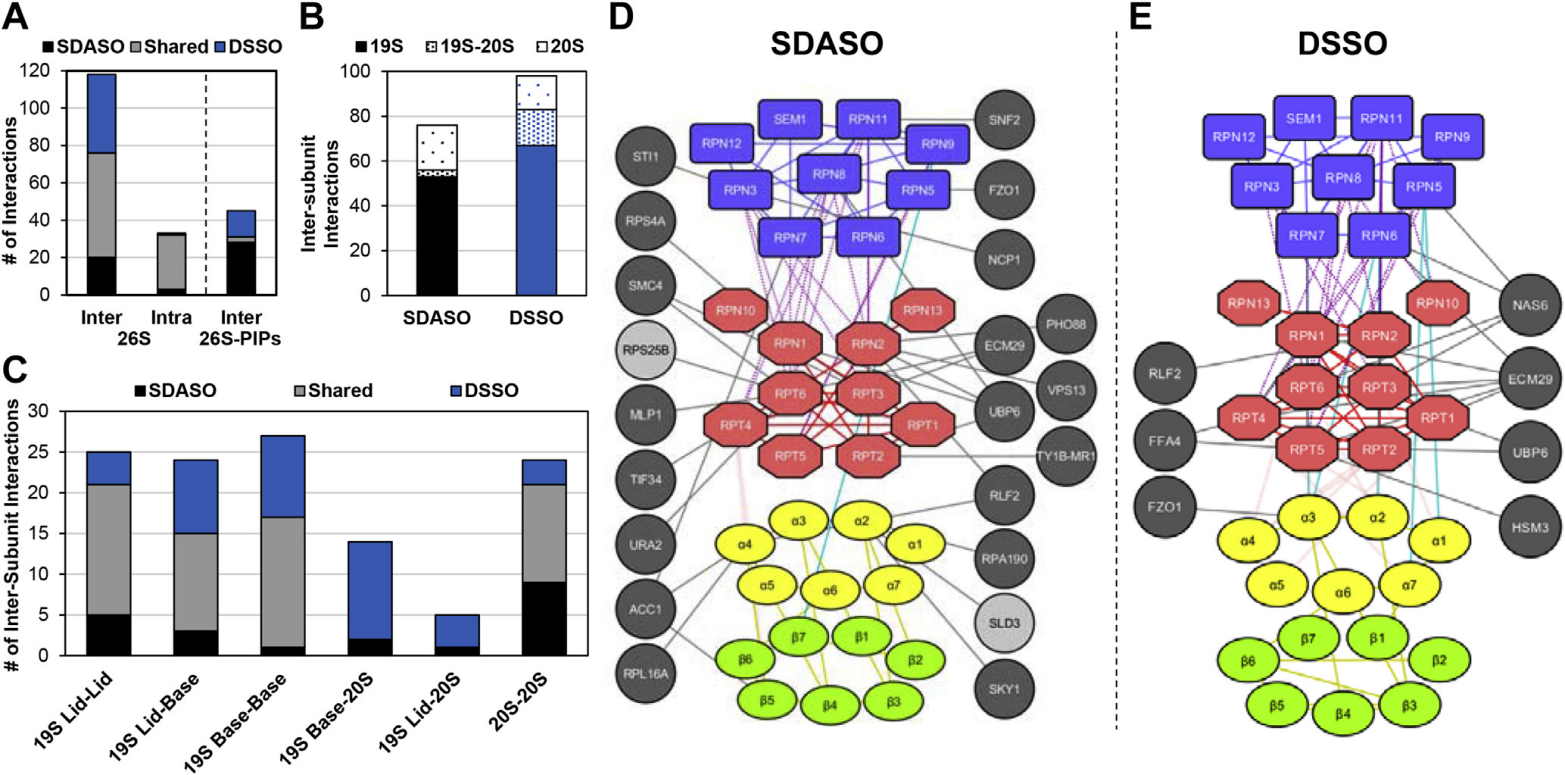
**XL-PPI Analysis of the Yeast 26S proteasome.***A*, comparison of the total number of SDASO and DSSO XL-PPIs (intersubunit and intrasubunit) within the 26S Proteasome itself and with proteasome-interacting proteins (PIPs). *B*, distribution of the total number of SDASO and DSSO intersubunit interactions within the 26S proteasome. *C*, comparison of the distribution of SDASO and DSSO XL-PPIs among the six types of intersubcomplex and intrasubcomplex interactions within the 26S proteasome. *D*, SDASO and (*E*) DSSO XL-PPI networks of the 26S proteasome and its interacting proteins, in which protein nodes are colored as follows: 19S lid subunits (*light blue*), 19S base subunits (*light red*), 20S α subunits (*light yellow*), 20S β subunits (*green*), known PIPs (*dark gray*), and novel PIPs (*light gray*). The edges are colored as: 19S lid-lid (*blue*), 19S base-base (*red*), 19S lid-base (*purple*), 19S lid-20S core (*cyan*), 19S base-20S core (*pink*), 20S-20S (*gold*), and 26S-PIP (*black*). DSSO, disuccinimidyl sulfoxide; PPIs, protein–protein interactions; SDASO, succinimidyl diazirine sulfoxide.

To delineate the interactions captured by residue-specific and nonspecific cross-linkers, we took the cross-links identified in at least two biological replicates from all of our XL-MS experiments and combined SDASO data for further comparison. As a result, we obtained a total of 2186 SDASO cross-links (959 intersubunit, 1227 intrasubunit) and 1098 DSSO cross-links (649 intersubunit, 449 intrasubunit) of the 26S proteasome (supplemental Table S3*C*). From this data, 2-D and 3-D XL-maps were generated, displaying extensive connectivity among proteasome subunits (Fig. 5, *A*–*C*). In comparison, the most noticeable differences in the XL-maps are the increased density of intersubunit cross-links within the 19S by DSSO (Fig. 5, *A* and *B*) and within the 20S by SDASO (Fig. 5, *A* and *C*). When combined, SDASO and DSSO cross-links yielded a total of 118 intersubunit (78 SDASO and 98 DSSO) and 33 intrasubunit (31 SDASO and 30 DSSO) protein–protein interactions of the 26S proteasome (Fig. 6*A*). While 85 interactions were shared by both types of linkers, 23 interactions were unique to SDASO (20 intersubunit and three intrasubunit), and 43 interactions were only mapped by DSSO (42 intersubunit and one intrasubunit). For the intersubunit interactions, 47% were identified by both SDASO and DSSO, whereas 17% and 36% were revealed uniquely by SDASO and DSSO, respectively. For further examination, three types of interactions within the 26S proteasome were categorized: 19S-19S, 19S-20S, and 20S-20S (Fig. 6*B*), and the 19S-containing interactions were further subdivided into 19S lid-lid, lid-base, base-base, 19S lid-20S, and 19S base-20S interactions (Fig. 6*C*). In addition, SDASO and DSSO XL-PPI networks of the 26S proteasome were derived based on their respective cross-links (Fig. 6, *D* and *E*). While both SDASO and DSSO uncovered mostly 19S-containing interactions, DSSO is more efficient than SDASO at defining these interactions, *i.e.*, 19S base-base (26 *versus* 17), 19S lid-base (21 *versus* 15), and 19S-20S (16 *versus* three) (Fig. 6, *C*–*E*). In contrast, SDASO captured more 20S-20S interactions with significantly more cross-link coverage (20 SDASO XL-PPIs from 262 K-X linkages *versus* 15 DSSO XL-PPIs from 37 K-K linkages) (Figs. 5*C* and 6, *B*–*E*). Apart from intersubunit interactions, 29 out of 33 intrasubunit interactions were captured by both types of linkers (Fig. 6*A*), but SDASO identified a greater amount of contact sites within proteasome subunits relative to DSSO (supplemental Fig. S18, *A* and *B*, supplemental Table S3*C*).

**Fig. 7:**
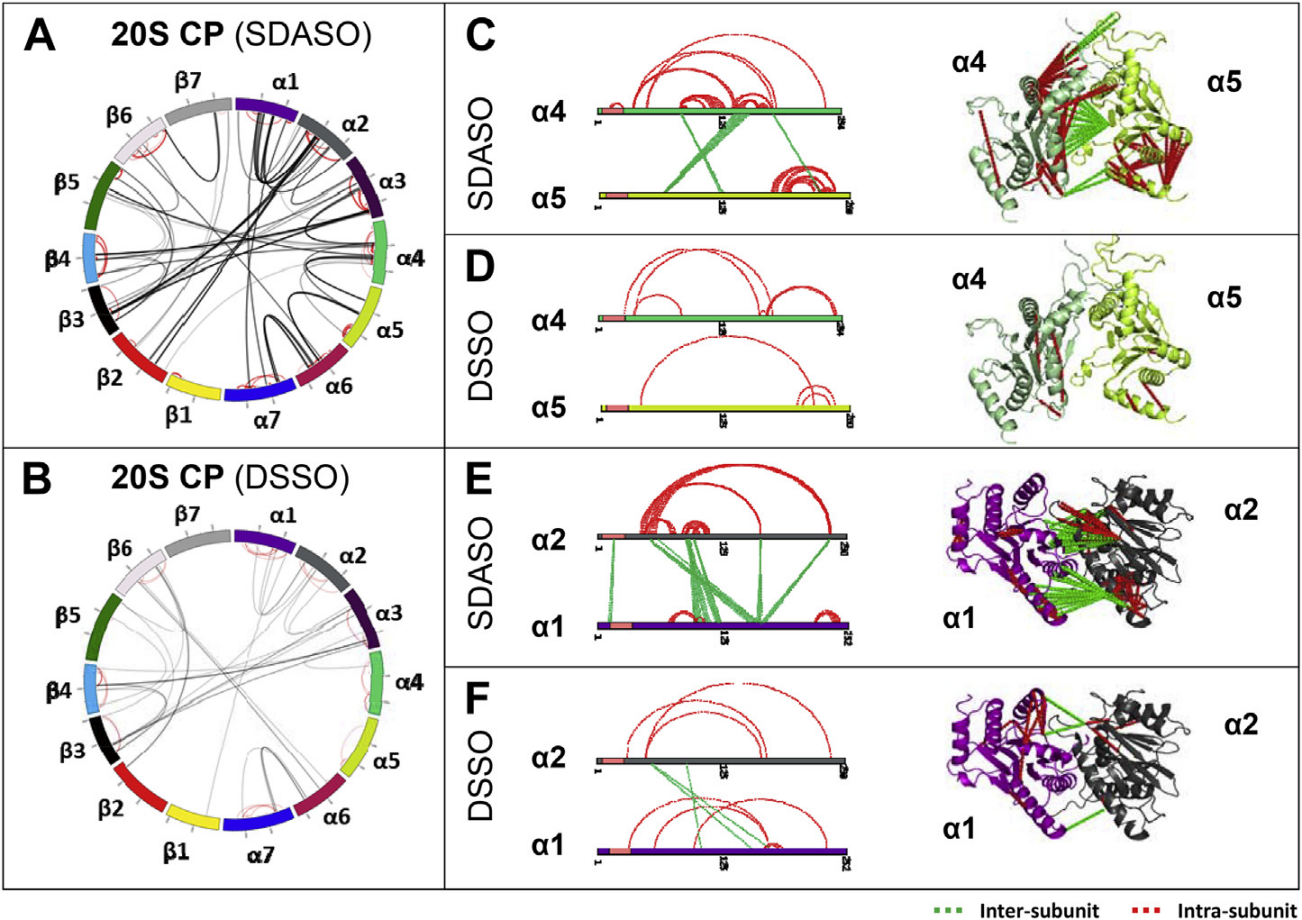
**SDASO and DSSO XL-maps of the 20S CP.***A*, circular 2-D SDASO XL-map of the 20S CP. *B*, circular 2-D DSSO XL-map of the 20S CP. 2-D and 3-D XL-maps of α4-α5 interaction based on (*C*) SDASO and (*D*) DSSO cross-links. 2-D and 3-D XL-maps of α1-α2 interaction based on (*E*) SDASO and (*F*) DSSO cross-links. Note: high-resolution structure of the yeast 26S proteasome (PDB: 4CR2 (s1)) was used here. For 2-D XL-maps, intersubunit linkages are colored *black* and intrasubunit linkages are colored *red*. For 3-D XL-maps, intersubunit linkages are shown in *green*, while intrasubunit linkages are *red*. CP, core particle; DSSO, disuccinimidyl sulfoxide; SDASO, succinimidyl diazirine sulfoxide.

In comparison, the 2-D XL-maps of the 20S CP demonstrate that SDASO provided broader coverage of both intersubunit and intrasubunit interactions (Fig. 7, *A* and *B*). For example, one of the intersubunit interactions uniquely identified by SDASO was between subunit α4 and α5, described by three contact regions (α5:K66-α4:X(147–162), α4:K88-α5:X(128–131), and α4:K182-α5:X(233–234)) (Fig. 7*C*). Although there are several lysine residues in α4 (K146, K169, and K177) and α5 (K32 and K52) proximal to the interfaces identified by SDASO, DSSO was not able to capture this particular interaction (Fig. 7*D*). While both SDASO and DSSO identified intrasubunit interactions of α4 and α5 that were complementary, SDASO yielded denser connectivity within each protein (Fig. 7, *C* and *D*). This type of observation is further exemplified by the intersubunit and intrasubunit interactions of α1 and α2 (Fig. 7, *E* and *F*). As shown, SDASO not only identified the same interaction regions as DSSO (*i.e.*, SDASO: α1:X(98–120)-α2:K91 and α1:X(157–168)-α2:K50 *versus* DSSO: α1:K107-α2:K91, α1:K167-α2:K50 and α1:K187-α2:K50) but also determined additional contacts (SDASO: α1:X(11–13)-α2:K17, α1:X(120–123)-α2:K98, α1:X(159–164)-α2:K166, and α1:X(161–166)-α2:K237). Taken together, these results indicate that SDASO is complementary to DSSO in mapping PPIs of protein complexes.

### Identification of Proteasome Interacting Proteins

Besides interactions within the 26S proteasome, we also examined physical contacts with co-purified proteasome-interacting proteins (PIPs). Considering only cross-links that were identified in at least two biological experiments from all of our XL-MS experiments, we obtained a total of 125 unique SDASO cross-linked peptides (175 K-X linkages) and 90 unique DSSO cross-linked peptides (90 K-K linkages), representing 44 interprotein and four intraprotein pair-wise interactions. This resulted in the identification of 24 PIPs (21 SDASO and seven DSSO) with direct contacts to the 26S proteasome, including 22 known (https://thebiogrid.org/) and two novel ones (Fig. 6, *D* and *E*), in which only four PIPs (Ecm29, Ubp6, Fzo1, and Rlf2) were found by both types of linkers (Fig. 6*A*, supplemental Table S3*C*). The four shared PIPs were identified with a total of 17 PPIs, of which only three (Rpt2-Ubp6, Rpt3-Ecm29, and Rpn2-Rlf2) were captured by both SDASO and DSSO. Among the known PIPs, Ecm29 is a key regulator of the 26S proteasome, and human Ecm29 has been shown to interact with Rpt1, Rpt4, Rpt5, Rpn1, and Rpn10 by DSSO cross-linking (54). Similarly, the interactions of yeast Ecm29 with Rpt1, Rpt4, Rpt5, and Rpn1 were confirmed by DSSO XL. In addition, Ecm29-Rpt3 and Ecm29-Rpn6 interactions from DSSO were identified for the first time. Furthermore, SDASO validated Ecm29-Rpt3 interaction and identified Ecm29-Rpt6 interaction (supplemental Fig. S19*A*). These results demonstrate extensive contacts between Ecm29 and the 26S proteasome, corroborating well with previous observation of its human orthologue (54). Ubp6 is a proteasome-associated deubiquitinase that interacts with the 26S proteasome through Rpn1 (55, 56). While DSSO caught Ubp6-Rpt1 and Ubp6-Rpt2 interactions as reported (56), SDASO identified extensive interactions of Ubp6 with multiple subunits including Rpn1, Rpn2, Rpn8, and Rpt2 (supplemental Fig. S19*B*). Overall, SDASO XL-MS analyses identified higher number of PIPs than DSSO, illustrating its capability of capturing interacting proteins in affinity purified samples.

### Relative Specificity of Diazirine Cross-Linking

Diazirine photoactivation leads to not only the production of reactive carbene for AA labeling through X-H bond insertion, but also isomerization to form diazo compound to specifically react with carboxyl groups (27). Recent studies have suggested that diazirine labeling shows preferences for acidic residues (27, 31). To examine this, we sought to determine whether any AA preference was observed in SDASO cross-linking of the 26S proteasome. On average, ~26% of residues cross-linked by SDASO linkers were determined precisely at a single site, whereas the rest were localized ambiguously at one out of two (~34%), three (~20%), or four and more (~20%) possible sites (supplemental Fig. S20*A*). Similar precisions in SDASO cross-linked site localization was also observed in BSA data (supplemental Fig. S20*B*), consistent with conventional diazirine linkers (29). To prevent overestimation due to site ambiguity, we calculated the weighted AA occurrence to assess the preference of diazirine labeling in the 26S proteasome, similarly as described (31) (See Experimental Procedures section). Our results suggest that glutamic acid was the most favored by diazirine cross-linking, representing ~30% of the targeted residues for all three SDASO linkers (supplemental Fig. S21*A*). In comparison, four additional residues, *i.e.*, alanine (7.2%), aspartic acid (6.8%), leucine (7.3%), and tyrosine (6.4%) were targeted relatively favorably by SDASOs, as they had an average frequency well above those of the remaining AAs (2.7%). The dominant preference of glutamic acid displayed by diazirine cross-linking in proteasome samples was also detected in BSA, in which ~25% of SDASO cross-linked sites were glutamic acids (supplemental Fig. S21*B*, supplemental Table S1*D*). Interestingly, five relatively favorable diazirine cross-linked sites in BSA contained aspartic acid, histidine, threonine, valine, and tyrosine with an average frequency of 6.8~8.4%, in which only aspartic acid and tyrosine residues showed similar preference in proteasome samples. This discrepancy is more likely attributed to the occurrence of common AAs in close proximity to cross-linkable lysines at interaction interfaces within proteins of interest as well as MS detectability and identification of the resulting cross-linked peptides. Nonetheless, while diazirine reactivity is nonspecific, our results suggest that it preferably targets a subset of AAs with glutamic acid as its most favored one.

## Discussion

Here, we report the development and characterization of three sulfoxide-containing MS-cleavable heterobifunctional photoactivated cross-linkers, SDASO-L, -M, and -S. While built upon our previously developed amine-reactive DSSO (33), SDASO cross-linkers are distinctly different, representing the first generation of sulfoxide-containing MS-cleavable heterobifunctional cross-linkers. The unique designs of the SDASO linkers enable a single labile bond to be preferentially cleaved over peptide backbone, leading to only one pair of MS^2^ fragment ions and enhancing analysis sensitivity (47). Importantly, SDASO cross-linked peptides possess robust and predictable MS^2^ fragmentation characteristics similar to sulfoxide-containing homobifunctional cross-linkers, thus permitting their fast and accurate identification using MS^n^-based XL-MS workflow (10, 12, 33, 34, 35). Although MS^2^-based approaches have been widely used in XL-MS studies (2), it is important to note that MS^n^ analysis is critical for effective database searching to identify photocross-linked peptides and localize nonspecific cross-linked sites with speed and accuracy, especially for complex samples. Owing to their unique capabilities, the SDASO cross-linkers have been successfully employed to study PPIs of not only a single protein BSA but also the affinity purified yeast 26S proteasome complex. To the best of our knowledge, this work represents the first application of photoactivated cross-linking on PPI mapping of large protein assemblies. The development of SDASO cross-linkers further demonstrates the robustness and potential of our XL-MS technology based on sulfoxide-containing MS-cleavable cross-linkers and provides a viable analytical platform for the expansion of new MS-cleavable reagents to generate a complete PPI map of cellular systems in the future.

Although photoinduced diazirine labeling is nonspecific, the observed reproducibility of cross-linked peptide sequences was comparable for SDASO and residue-specific cross-linkers (8), supporting the reliability of photoactivated cross-linked products. While all of the 20 common AAs were detected as SDASO cross-linked sites in this work, SDASO displays preferential labeling of glutamic acids, corroborating well with previous reports on diazirine favoring acidic residues (27, 31). Although aspartic acids are in comparable abundance to glutamic acids in BSA and proteasomes, they were targeted noticeably less by SDASO. In comparison, acidic residue-reactive cross-linkers such as DHSO do not appear to have noticeable differences in reactivity toward these two AAs (8, 10). Therefore, the preferential labeling of glutamic acids over aspartic acids displayed by diazirine may be because of differences in physiochemical properties of their side-chains and short-lived photoactivated reaction. In addition to acidic residues, several AAs including tyrosine, valine, leucine, threonine, and histidine have been detected as SDASO cross-linked sites more often than other AAs, in which tyrosine and histidine residues have exhibited favored carbene insertion in the past (31). The preferred reactivity of SDASO cross-linkers toward a subset of AAs including ones that cannot be easily targeted by specific cross-linking chemistries is beneficial to XL-MS studies, as it helps enhance the analysis of the resulting photoactivated cross-linked peptides and expand PPI coverage.

The complementarity in PPI mapping among the three SDASO linkers appears to be much more pronounced in the XL-MS analyses of proteasomes than BSA, implying the benefits of variable linker lengths for complex PPI profiling. In comparison to residue-specific cross-linkers such as DSSO, DHSO, and BMSO (10, 12), SDASO XL-MS analyses of BSA has yielded the highest number of cross-linked peptides and the most comprehensive interaction maps. The high-density SDASO XL-maps of BSA illustrates the effectiveness of the heterobifunctional photocross-linkers for mapping a diverse range of interactions, which is in good agreement with previous reports (25, 26). Intriguingly, while SDASO XL-MS analysis of the yeast 26S proteasome identified extensive intersubunit and intrasubunit interactions, the overall scopes of PPIs obtained from all three SDASO linkers is only comparable to those by DSSO and other residue-specific cross-linkers (53). Although DSSO produced a higher number of cross-linked peptides of the 26S proteasome than SDASO, comparisons of their cross-linked peptide sequences have revealed limited overlaps. Owing to diazirine nonspecificity, SDASO XL-maps of the 26S proteasome contain much more residue-to-residue connectivity. In addition, the three SDASO linkers have captured more interactions of the stable and compact 20S CP, but less of the dynamic and flexible 19S RP than DSSO. Because the spacer arm lengths of DSSO and SDASO linkers are similar, variance in PPI coverages is mostly attributed to cross-linkers’ reactivity and kinetics (29). Collectively, our results have demonstrated the value of SDASO photocross-linkers in probing PPIs of both simple and complex samples. The extensive SDASO XL-MS data have allowed us not only to obtain comprehensive XL-maps complementary to those of existing cross-linkers but more importantly to better assess the reliability and capability of diazirine cross-linking in probing PPIs. Therefore, this work has established a solid foundation for future applications of photocross-linking in complex XL-MS studies.

## Data Availability

Raw data have been deposited at the PRIDE Archive proteomics data repository (ID: PXD022690). Annotated spectra for cross-link identifications can be viewed through MS-Viewer (https://msviewer.ucsf.edu/prospector/cgi-bin/msform.cgi?form=msviewer) using the provided links in the supplemental data.

## Supplemental data

This article contains supplemental data (10, 12, 33, 57, 58, 59).

## Conflict of interest

The authors declare no conflict of interest.

## Supplemental Data

Supplemental Table S1

Supplemental Table S2A

Supplemental Table S2B

Supplemental Table S2C

Supplemental Table S3A–C
