# Supplementary material for: Enabling Photoactivated Cross-Linking Mass Spectrometric Analysis of Protein Complexes by Novel MS-Cleavable Cross-Linkers
Source: Mol Cell Proteomics. 2021 Apr 27;20:100084. doi: 10.1016/j.mcpro.2021.100084 (PMC8214149; doi:10.1016/j.mcpro.2021.100084)
Supplement: Supplementary file 1 — Supplemental Materials and Figures S1–S21 [file mmc1.pdf]

SUPPLEMENTAL INFORMATION for

Enabling Photoactivated Cross-linking Mass Spectrometric Analysis of Protein Complexes by Novel MS-cleavable Cross-linkers

Craig Gutierrez[1], Leah J. Salituro[2], Clinton Yu[1], Xiaorong Wang[1], Sadie F. Depeter[2], Scott D. Rychnovsky[2], Lan Huang[1]*

[1]Department of Physiology and Biophysics, University of California, Irvine, CA 92697

[2]Department of Chemistry, University of California, Irvine, CA 92697

*Correspondence should be addressed to Dr. Lan Huang (lanhuang@uci.edu)

Medical Science I, D233

Department of Physiology & Biophysics

University of California, Irvine

Irvine, CA 92697-4560

Phone: (949) 824-8548

Fax: (949) 824-8540

SUPPLEMENTAL METHODS

Materials and Reagents

All chemicals were purchased from Aldrich, Acros Organics, Alfa Aesar, TCI, VWR Internationa or Fisher Scientific and used without further purification. Deuterated solvents were purchased from Cambridge Isotope Laboratories. Solvents were purchased as ACS grade or better and as HPLC-grade and passed through a solvent purification system equipped with activated alumina columns prior to use.

Synthesis and Characterization of SDASO Cross-linkers

All reactions were carried out in oven-dried glassware, under an atmosphere of argon unless otherwise noted. Reactions were monitored by thin layer chromatography (TLC) or electrospray ionization mass spectrometry (ESI-MS). Thin layer chromatography (TLC) was carried out using glass plates coated with a 250 μm layer of 60 Å silica gel. TLC plates were visualized with a UV lamp at 254 nm, or by staining with potassium permanganate, cerium molybdate, or ninhydrin. ESI-MS was analyzed in positive mode with flow injection. Liquid chromatography was performed using a Teledyne ISCO CombiFlash® forced flow with an automated purification system on prepacked silica gel (SiO_2) columns or prepacked C18 columns. Proton NMRs were recorded at 500 MHz or 600 MHz using either a Bruker DRX500 (cryoprobe) or a Bruker AVANCE600 (cryoprobe) NMR, respectively. Carbon NMRs were recorded at 126 MHz or 151 MHz on the Bruker DRX500 or Bruker AVANCE600 NMR, respectively. All NMRs were taken at 25 °C. Chemical shifts (δ) are reported in parts per million (ppm) and referenced to residual solvent peak at 7.26 ppm (^1H) or 77.16 ppm (^{13}C) for deuterated chloroform ($CDCl_3$), 2.50 ppm (^1H) or 39.52 ppm (^{13}C) for deuterated dimethyl sulfoxide (DMSO-d_6), 3.31 ppm (^1H) or 49.00 (^{13}C) for deuterated methanol (CD_3OD). NMR data are reported as the following: chemical

shift, multiplicity (s = singlet, d = doublet, t = triplet, q = quartet, m = multiplet, br = broad), coupling constants (J) in hertz (Hz), and integration. High-resolution mass spectrometry (HRMS) was performed using a Waters LCT Premier TOF spectrometer with ESI source.

1. SDASO-S

2-(3-Methyl-3*H*-diazirin-3-yl)ethyl 4-methylbenzenesulfonate (S1)

As shown in Fig. S1a, the staring material, 4-hydroxy-2-butanone (3.0 mL, 34.5 mmol, 1.0 equiv), was cooled to 0 °C and then 7N NH_3 in MeOH (35 mL) was added dropwise. After stirring for 3 h at 0 °C, a solution of hydroxylamine-*O*-sulfonic acid (H_2NOSO_3H; 4.25 g, 37.5 mmol, 1.1 equiv) in MeOH (15 mL) was added dropwise then solution was allowed to warm to rt. After stirring for 17 h the reaction solution was concentrated *in vacuo*. Next, NEt_3 (7.5 mL, 53.8, 1.6 equiv) was added to a solution of the residue in MeOH (25 mL) at 0 °C. After stirring for 10 min at 0 °C, iodine was added in aliquots until a dark brown color persisted in the solution. The reaction solution was diluted in EtOAc, washed with 1.0 M aq. HCl, sat. aq. $Na_2S_2O_3$ solution, and brine. Finally, the combined organic phase was dried over $MgSO_4$ and concentrated *in vacuo*. The resulting residue was subject to the next reaction without further purification.

This crude product was dissolved in pyridine (10.0 mL, 124 mmol, 3.6 equiv) and cooled to 0 °C. Next, *p*-toluenesulfonyl chloride (TsCl; 1.86 g, 10.0 mmol, 0.3 equiv) was added to the solution. After stirring for 1 h at rt, the reaction solution was diluted with EtOAc, washed with 1N HCl, sat. aq. $NaHCO_3$ solution, and brine. The combined organic phase was then dried over $MgSO_4$ and concentrated *in vacuo*. The resulting residue was purified by flash chromatography (15% EtOAc in hexanes) to obtain tosylate **S1** (971 mg, 11% over two steps). **S1**: ^{1}H NMR (500 MHz, $CDCl_3$): δ 7.82 (d, *J* = 8.5 Hz, 2H), 7.36 (d, *J* = 8.5 Hz, 2H), 3.96 (t, *J* = 6.4 Hz, 2H), 2.46 (s, 3H), 1.67 (t, *J* = 6.4 Hz, 2H), 1.00 (s, 3H); ^{13}C NMR (126 MHz, $CDCl_3$): δ 145.2, 132.9, 130.1, 128.1,

65.2, 34.3, 23.5, 21.8, 19.9. Spectral data were consistent with those previously reported for the compound (1)

Methyl 3-((2-(3-methyl-3*H*-diazirin-3-yl)ethyl)thio)propanoate (S2)

As shown in Fig. S1a, K_2CO_3 (477 mg, 3.5 mmol, 1.5 equiv) was added to a solution of tosylate **S1** (579 mg, 2.3 mmol, 1.0 equiv,) and methyl-3-mercaptopropionate (0.50 mL, 4.6 mmol, 2 equiv) in MeOH (2.3 mL). After stirring for 3 h at rt, the reaction solution was filtered to remove solids. The filtrate was concentrated *in vacuo* and the resulting residue was purified by flash chromatography (20% EtOAc in hexanes) to obtain ester **S2** (408 mg, 88%). **S2**: ^{1}H NMR (500 MHz, $CDCl_3$): δ 3.70 (s, 3H), 2.73 (t, J = 7.4 Hz, 2H), 2.56 (t, J = 7.4 Hz, 2H), 2.35 (t, J = 8.0 Hz, 2H), 1.61 (t, J = 8.0 Hz, 2H), 1.02 (s, 3H); ^{13}C NMR (126 MHz, $CDCl_3$): δ 172.3, 51.9, 34.64, 34.61, 27.1, 26.6, 25.3, 19.9; HRMS (ESI, MeOH) *m/z*: $[M+Na]^+$ calcd for $C_8H_{14}N_2O_2SNa$ 225.0669, found 225.0677.

3-((2-(3-Methyl-3*H*-diazirin-3-yl)ethyl)thio)propanoic acid (S3)

As shown in Fig. S1a, $LiOH{\cdot}H_2O$ (79 mg, 1.9 mmol, 1.0 equiv) was added to a solution of ester **S2** (382 mg, 1.9 mmol, 1.0 equiv) in THF:H_2O (4:1, 12.7 mL). After stirring at rt for 1 h, an additional aliquot of $LiOH{\cdot}H_2O$ (79 mg, 1.9 mmol, 1.0 equiv) was added. After stirring for an additional 2 h at rt, the reaction mixture was partitioned between hexanes and H_2O. The aqueous phase was acidified to pH = 1 with 1N HCl and extracted with EtOAc five times. The combined organic layers were dried with $MgSO_4$, and concentrated *in vacuo*. The crude product (acid **S3**) was subjected to the next step without further purification.

2,5-Dioxopyrrolidin-1-yl 3-((2-(3-methyl-3*H*-diazirin-3-yl)ethyl)thio)propanoate (S4)

As shown in Fig. S1a, *N*-hydroxysuccinimide (NHS-H; 219 mg, 1.9 mmol, 1.0 equiv) was added to a solution of acid **S3** (358 mg, 1.9 mmol, 1.0 equiv) in CH_2Cl_2 (4.0 mL) followed by an

addition of 1-(3-dimethylaminopropyl)-3-ethylcarbodiimide hydrochloride (EDC·HCl; 401 mg, 2.1 mmol, 1.1 equiv). After stirring at rt for 16 h, the reaction solution was diluted in CH_2Cl_2, washed with H_2O, then brine. The combined organic phase was dried with $MgSO_4$ and concentrated *in vacuo*. The resulting residue was purified by flash chromatography (50% EtOAc in hexanes) to obtain NHS ester **S4** (308 mg containing 5 wt% EtOAc by ^{1}H NMR, 292 mg, 54% over two steps). **S4**: ^{1}H NMR (500 MHz, $CDCl_3$): δ 2.93–2.78 (m, 8H), 2.42 (t, *J* = 8.0, 2H), 1.65 (t, *J* = 8.0, 2H), 1.05 (s, 3H); ^{13}C NMR (126 MHz, $CDCl_3$): δ 169.0, 167.2, 34.6, 32.1, 26.9, 26.6, 25.7, 25.3, 19.9.

2,5-Dioxopyrrolidin-1-yl 3-((2-(3-methyl-3*H*-diazirin-3-yl)ethyl)sulfinyl)propanoate (SDASO-S)

Finally, as shown in Fig. S1a, 30% aq. H_2O_2 (72 µL, 0.70 mmol, 2.0 equiv) was added to a solution of NHS ester **S4** (100 mg, 0.35 mmol, 1.0 equiv) in 1,1,1,3,3,3-hexafluoro-2-propanol (HFIP; 0.9 mL) was added. After stirring at rt for 20 min, the reaction was quenched with dimethyl sulfide (DMS; 0.15 mL) and the mixture was allowed to stir for an additional 10 min. The reaction mixture was concentrated *in vacuo*, then partitioned between $CHCl_3$ and H_2O and the aqueous phase was extracted $CHCl_3$. The combined organic phase was washed with H_2O and the combined aqueous phase was again extracted with $CHCl_3$. The combined organic phase was washed with brine, dried with $MgSO_4$, and concentrated *in vacuo* to obtain **SDASO-S** as a white solid (75 mg, 93% purity by ^{1}H NMR, 70 mg, 66%). **SDASO-S**: ^{1}H NMR (500 MHz, $CDCl_3$): δ 3.21–3.05 (m, 3H), 3.01–2.91 (m, 1H), 2.84 (s, 4H), 2.65–2.56 (m, 1H), 2.56–2.47 (m, 1H), 1.95–1.79 (m, 2H), 1.09 (s, 3H); ^{13}C NMR (126 MHz, $CDCl_3$): δ 168.8, 167.1, 47.0, 46.2, 28.0, 25.7, 24.9, 24.2, 19.8; HRMS (ESI, MeOH) *m/z*: $[M+Na]^+$ calcd for $C_{11}H_{15}N_3O_5SNa$ 324.0625, found 324.0636.

2. SDASO-M

3-(3-Methyl-3*H*-diazirin-3-yl)propyl 4-methylbenzenesulfonate (S5)

As shown in Fig. S1b, the staring material, 5-hydroxy-2-pentanone (1.5 mL, 14.6 mmol, 1.0 equiv), was cooled to 0 °C and 7N NH_3 in MeOH (15 mL) was added dropwise. After stirring for 3 h at 0 °C, a solution of H_2NOSO_3H (1.90 g, 16.8 mmol, 1.15 equiv) in MeOH (12.4 mL) was added dropwise and the solution was allowed to warm to rt. After stirring for 17.5 h the reaction solution was concentrated *in vacuo*. Next, NEt_3 (3.3 mL, 23.4, 1.6 equiv) was added to a solution of the residue in MeOH (15 mL) at 0 °C. After stirring for 10 min at 0 °C, iodine was added in aliquots until a dark brown color persisted in the solution. The reaction solution was diluted in EtOAc, washed with 1.0 M aq. HCl, sat. aq. $Na_2S_2O_3$ solution, and brine. The combined organic phase was dried over $MgSO_4$ and concentrated *in vacuo*. The resulting residue was subject to the next reaction without further purification.

This crude product was dissolved in pyridine (4.2 mL, 52.6 mmol, 3.6 equiv) and cooled to 0 °C. Next, TsCl (835 mg, 4.38 mmol, 0.3 equiv) was added to the solution. After stirring for 1 h at rt, the reaction solution was diluted with EtOAc, washed with 1N HCl, sat. aq. $NaHCO_3$ solution, and brine. The combined organic phase was dried over $MgSO_4$ and concentrated *in vacuo*. The resulting residue was purified by flash chromatography (0% to 15% EtOAc in hexanes) to obtain tosylate **S5** (102 mg, 3% over two steps). **S5**: ^{1}H NMR (500 MHz, $CDCl_3$): δ 7.75 (d, *J* = 8.2 Hz, 2H), 7.33 (d, *J* = 8.0 Hz, 2H), 3.97 (t, *J* = 6.2 Hz, 2H), 2.43 (s, 3H), 1.55–1.42 (m, 2H), 1.42–1.31 (m, 2H), 0.95 (s, 3H); ^{13}C NMR (126 MHz, $CDCl_3$): δ 145.0, 133.0, 130.0, 127.9, 69.5, 30.4, 25.1, 23.6, 21.7, 19.8.

Methyl 3-((3-(3-methyl-3*H*-diazirin-3-yl)propyl)thio)propanoate (S6)

As shown in Fig. S1b, K_2CO_3 (79 mg, 0.57 mmol, 1.5 equiv) was added to a solution of tosylate **S5** (102 mg, 0.38 mmol, 1.0 equiv,) and methyl-3-mercaptopropionate (84 µL, 0.76 mmol,

2 equiv) in MeOH (0.4 mL). After stirring for 3 h at rt, the reaction mixture was filtered to remove solids. The filtrate was concentrated *in vacuo* and the resulting residue was purified by flash chromatography (0% to 20% EtOAc in hexanes) to obtain ester **S6** (95 mg, containing 6 wt% of EtOAc and 17 wt% methyl-3-mercaptopropionate by ^{1}H NMR; 73 mg, 89%). **S6**: ^{1}H NMR (500 MHz, $CDCl_3$): δ 3.69 (s, 3H), 2.81–2.72 (m, 2H), 2.59 (t, *J* = 7.3 Hz, 2H), 2.53–2.45 (m, 2H), 1.51–1.41 (m, 2H), 1.00 (s, 3H).

3-((3-(3-Methyl-3*H*-diazirin-3-yl)propyl)thio)propanoic acid (S7)

As shown in Fig. S1b, $LiOH{\cdot}H_2O$ (16 mg, 0.38 mmol, 1.05 equiv) was added to a solution of ester **S6** (73 mg, 0.36 mmol, 1.0 equiv) in THF:H_2O (4:1, 2.5 mL). After stirring at rt for 1 h, an additional aliquot of $LiOH{\cdot}H_2O$ (16 mg, 0.38 mmol, 1.05 equiv) was added. After stirring for an additional 2 h at rt, the reaction mixture was partitioned between hexanes and H_2O. The aqueous phase was acidified to pH = 1 with 1N HCl and extracted with EtOAc five times. The combined organic layers were dried with $MgSO_4$, and concentrated *in vacuo*. The crude product (acid **S7**) was subjected to the next step without further purification.

2,5-Dioxopyrrolidin-1-yl 3-((3-(3-methyl-3*H*-diazirin-3-yl)propyl)thio)propanoate (S8)

As shown in Fig. S1b, NHS-H (40 mg, 0.35 mmol, 1.0 equiv) was added to a solution of crude acid **S7** (70 mg, 0.35 mmol, 1.0 equiv) in CH_2Cl_2 (0.7 mL) followed by an addition of EDC·HCl (74 mg, 0.39 mmol, 1.1 equiv). After stirring at rt for 15 h, the reaction solution was diluted in CH_2Cl_2, washed with H_2O, then brine. The combined organic phase was dried with $MgSO_4$ and concentrated *in vacuo* to obtain crude NHS ester **S8** (56 mg). **S8**: ^{1}H NMR (500 MHz, $CDCl_3$): δ 2.95–2.76 (m, 8H), 2.54–2.41 (m, 2H), 1.48–1.37 (m, 4H), 0.99 (s, 3H); ^{13}C NMR (126 MHz, $CDCl_3$): δ 169.1, 167.2, 33.3, 32.1, 31.6, 26.3, 25.7, 25.5, 24.0, 19.9.

2,5-Dioxopyrrolidin-1-yl 3-((3-(3-methyl-3*H*-diazirin-3-yl)propyl)sulfinyl)propanoate (SDASO-M)

Finally, as shown in Fig. S1b, 30% aq. H_2O_2 (38 μL, 0.37 mmol, 2.0 equiv) was added to a solution of crude NHS ester **S8** (56 mg, 0.19 mmol, 1.0 equiv) in HFIP (0.5 mL). After stirring at rt for 20 min, the reaction was quenched with dimethyl sulfide (DMS; 80 μL) and the solution was allowed to stir for an additional 10 min. The reaction mixture was concentrated *in vacuo*, then partitioned between $CHCl_3$ and H_2O and the aqueous phase was extracted $CHCl_3$. The combined organic phase was washed with H_2O and the combined aqueous phase was again extracted with $CHCl_3$. The combined organic phase was washed with brine, dried with $MgSO_4$, and concentrated *in vacuo* to obtain crude **SDASO-M** (15 mg,). **SDASO-M**: ^{1}H NMR (500 MHz, $CDCl_3$): δ 3.23–2.92 (m, 4H), 2.85 (s, 4H), 2.77–2.57 (m, 2H), 1.82–1.62 (m, 2H), 1.62–1.43 (m, 2H), 1.04 (s, 3H); ^{13}C NMR (126 MHz, $CDCl_3$): δ 168.8, 167.2, 51.7, 46.0, 33.4, 25.72, 25.69, 24.2, 19.7, 17.8.

3. SDASO-L

3-(3-Methyl-3*H*-diazirin-3-yl)propanoic acid (S9)

As shown in Fig. S1c, 7N NH_3 in MeOH (14.8 mL) was added to a solution of levulinic acid (2.0 mL, 19.5 mmol, 1.0 equiv) in MeOH (4.5 mL, 4.30 M) cooled to 0 °C. After stirring for 3 h at 0 °C, a solution of H_2NOSO_3H (2.54 g, 22.4 mmol, 1.15 equiv) in MeOH (13 mL) was added and the reaction was allowed to warm to rt. The milky white solution was concentrated *in vacuo* after stirring for 19 h. Next, NEt_3 (4.7 mL, 33.6 mmol, 1.5 equiv) was added to a solution of the white oil in MeOH (14 mL) at 0 °C. After stirring for 10 min at 0 °C, iodine was added in aliquots (10.2 g, 40.3 mmol, 1.8 equiv) until a dark brown color persisted in the solution. The brown mixture was diluted in EtOAc, washed with 1.0 M aq. HCl, sat. aq. $Na_2S_2O_3$ solution, and brine. The aqueous phases were combined and extracted three times with EtOAc. The combined organic

phase was dried over Na_2SO_4 and concentrated *in vacuo* to obtain a yellow solid which was subject to the next reaction without further purification. Spectral data were consistent with those previously reported for the compound (2).

2,5-Dioxopyrrolidin-1-yl 3-(3-methyl-3*H*-diazirin-3-yl)propanoate (S10)

As shown in Fig. S1c, trifluoroacetic anhydride (TFAA; 4.1 mL, 29.4 mmol, 2.0 equiv) was added dropwise to a solution of acid **S9** (1.88 g, 14.7 mmol, 1.0 equiv), NHS-H (3.38 g, 29.4 mmol, 2.0 equiv), and *N*,*N*-diisopropylethylamine (DIPEA; 10.2 mL, 58.7 mmol, 4.0 equiv) in DMF (75.0 mL, 0.20 M) cooled to 0 °C. The orange solution was stirred for 3 h at 0 °C, and then partitioned between EtOAc and brine. The organic phase was washed with brine five times, dried over Na_2SO_4, and concentrated *in vacuo* to obtain an orange oil. The oil was purified by flash chromatography (20 to 70% EtOAc in hexanes) to obtain NHS ester **S10** as a tan powder (1.65 g, 50% over two steps). Spectral data were consistent with those previously reported for the compound(2).

***N*-(2-mercaptoethyl)-3-(3-methyl-3*H*-diazirin-3-yl)propenamide (S11)**

As shown in Fig. S1c, NHS ester **S10** (1.00 g, 4.44 mmol, 1.0 equiv) was added to a solution of 2-aminoethanethiol (343 mg, 4.44 mmol, 1.0 equiv) in CH_2Cl_2 (22.2 mL, 0.20 M). After stirring for 20 min at rt, the reaction solution was concentrated *in vacuo*. The crude product (thiol **S11**) was subject to the next step without further purification.

2,5-Dioxopyrrolidin-1-yl acrylate (S12)

As shown in Fig. S1c, NEt_3 (6.30 mL, 45.0 mmol, 1.0 equiv) was added to a solution of NHS-H (5.18 g, 45.0 mmol, 1.0 equiv) in CH_2Cl_2 (90 mL, 0.50 M) cooled to 0 °C followed by an addition of acryloyl chloride (4.0 mL, 49.5 mmol, 1.1 equiv). The reaction was stirred for 3 h at 0 °C, and then vacuum filtered to collect the white precipitate. The filtrate was washed with H_2O

then brine, dried over Na_2SO_4, and concentrated *in vacuo* to obtain NHS ester **S12** as a white solid (5.52 g, 72%). Spectral data were consistent with those previously reported for the compound (3).

2,5-Dioxopyrrolidin-1-yl 3-((2-(3-(3-methyl-3*H*-diazirin-3-l) propanamido)ethyl)thio)propanoate (S13)

As shown in Fig. S1c, a solution of thiol **S11** (832 mg, 4.44 mmol, 1.0 equiv) in CH_2Cl_2 (22.2 mL, 0.20 M) was added to NHS ester **S12** (751 mg, 4.44 mmol, 1.0 equiv) followed by an addition of NEt_3 (0.680 mL, 4.88 mmol, 1.1 equiv). After stirring at rt for 20 min, the reaction solution was washed with H_2O then brine, dried with Na_2SO_4, and concentered *in vacuo*. The crude mixture was purified by flash chromatography (20 to 100% EtOAc in hexanes) to obtain sulfide **S13** (887 mg, 56% over two steps). **S13**: ^{1}H NMR (500 MHz, $CDCl_3$): δ 6.32 (s, br, 1H), 3.42 (q, *J* = 6.0 Hz, 2H), 2.96–2.80 (m, 8H), 2.70 (t, *J* = 6.0 Hz, 2H), 2.00 (t, *J* = 7.7 Hz, 2H), 1.72 (t, *J* = 7.7 Hz, 2H), 1.00 (s, 3H); ^{13}C NMR (125 MHz, $CDCl_3$): δ 171.7, 169.2, 167.3, 38.4, 32.4, 32.2, 30.6, 30.1, 26.5, 25.7, 25.6, 20.0; HRMS (ESI, MeOH) *m/z* calcd for $C_{14}H_{20}N_4O_5SNa$ $(M+Na)^+$ 379.1047, found 379.1043.

2,5-Dioxopyrrolidin-1-yl 3-((2-(3-(3-methyl-3*H*-diazirin-3-yl)propanamido)ethyl)sulfinyl) propanoate (SDASO-L)

Finally, as shown in Fig. S1c, 30% aq. H_2O_2 solution (0.11 mL, 1.11 mmol, 2.0 equiv) was added to a solution of sulfide **S12** (200 mg, 0.561 mmol, 1.0 equiv) in HFIP (2.80 mL, 0.20 M). The reaction was stirred for 10 min at rt, and then quenched with DMS (0.20 mL) and the mixture was concentrated *in vacuo* to obtain the cross linker **SDASO** (247 mg containing 23 wt% HFIP and 4 wt% DMSO; 181 mg, 87%). **SDASO**: ^{1}H NMR (600 MHz, $CDCl_3$): δ 6.77 (s, br, 1H), 3.85–3.65 (m, 2H), 3.20–3.04 (m, 4H), 2.95–2.80 (m, 4H), 2.63 (s, 4H), 2.04 (t, *J* = 7.6 Hz, 2H), 1.73 (t, *J* = 7.6 Hz, 2H), 1.01 (s, 3H); ^{13}C NMR (151 MHz, $CDCl_3$): δ 172.6, 168.9, 167.0, 51.3, 45.9,

34.4, 30.6, 30.0, 25.7, 25.6, 24.3, 19.9; HRMS (ESI, MeOH) *m/z* calcd for $C_{14}H_{20}N_4O_6SNa$ $(M+Na)^+$ 395.0996, found 395.0995.

SUPPLEMENTAL TABLES

Supplemental Table 1A. Detailed Summary of Cross-linked Peptides of BSA Identified for SDASO-L, -M, and -S Cross-linkers

Supplemental Table 1B. Summary of the Unique SDASO K-X Linkages of BSA

Supplemental Table 1C. Distance Mapping of SDASO K-X Linkages onto the BSA Structure (PDB: 4F5S)

Supplemental Table 1D. The Observed Frequency of Diazirine Labeling on Each AAs in BSA

Supplemental Table 2A. Detailed Summary of Cross-linked Peptides of the Yeast 26S Proteasome and its PIPs Identified for SDASO (-L, -M and -S) and DSSO Cross-linkers

Supplemental Table 2B. Summary of the Unique SDASO K-X Linkages of the Yeast 26S Proteasome and PIPs

Supplemental Table 2C. Summary of the Unique DSSO K-K Linkages of the Yeast 26S Proteasome and PIPs

Supplemental Table 3A. Cross-link Distance Mapping onto the Four Structural Models of the Yeast 26S Proteasome (s1-s4)

Supplemental Table 3B. The Observed Frequency of Diazirine Labeling on Each AAs in the Yeast 26S Proteasome

Supplemental Table 3C. XL-PPIs of the Yeast 26S Proteasome Derived from SDASO and DSSO Cross-linking

SUPPLEMENTAL FIGURE LEGENDS

Supplemental Figure 1. Synthesis pathways of the SDASO linkers: (**A**) SDASO-S. (**B**) SDASO-M. (**C**) SDASO-L.

Supplemental Figure 2. MS^2 fragmentation characteristics of SDASO linkers. (**A**) The conversion of sulfenic acid modified fragment α_S to unsaturated thiol modified fragment α_T. During MS^2-CID analysis, the sulfenic acid moiety loses water (-H_2O) to form the more stable unsaturated thiol (T) moiety, which is often detected as the dominant form. (b-c) Predicted MS^2 fragmentation of SDASO -L, -M and -S dead-end modified peptides (α_{DN}). Two types of dead-end products can be formed for SDASO linkers, depending on which reactive group is hydrolyzed. The products (α_{DN}) with a hydrolyzed NHS ester (**B**) or a hydrolyzed diazirine end (**C**) are illustrated. During MS^2-CID, the former only generates a thiol modified fragment α_T, whereas the latter only produces an alkene modified fragment α_A. (**D**) Predicted MS^2 fragmentation of SDASO-L, -M and -S intra-linked peptides (α_{intra}), yielding one fragment containing both alkene and thiol modifications (α_{A+T}).

Supplemental Figure 3. MS^n analyses of representative SDASO -L, -M and -S dead-end modified peptides of BSA. MS^2 spectra of the diazirine dead-end modified peptides α_{DN}: (**A**) SDASO-L: m/z 499.5673^{3+}, (**B**) SDASO-M: m/z 480.2411^{3+}, (**C**) SDASO-S: m/z 475.8982^{3+}. MS^2 spectra of the NHS ester dead-end modified peptides α_{DN} of BSA: (**D**) SDASO-L: m/z 765.0415^{3+}, (**E**) SDASO-M: m/z 745.7052^{3+}; (**F**) SDASO-S: m/z 741.0337^{3+}. MS^3 spectra of (**G**) α_A (m/z 652.32^{2+}) detected in (**A**) and (**J**) α_T (m/z 734.70^{2+}) detected in (**D**) from SDASO-L dead-end modified peptides. MS^3 spectra of (**H**) α_A (m/z 652.32^{2+}) detected in (**B**), and α_T (m/z 715.69^{2+}) detected in (**E**) from SDASO-M dead-end modified peptides. MS^3 spectra of (**H**) α_A (m/z 652.32^{2+}) detected in (**C**), and α_T (m/z 711.02^{2+}) detected in (**F**) from SDASO-S dead-end modified peptides.

The diazirine dead-end modified peptides of BSA were determined as ^{35}FKDLGEEHFK^{44}, in which K36 was modified for all SDASO linkers (**G-I**). The NHS ester dead-end modified peptides of BSA were identified as ^{168}RHPYFYAPELLYYANK^{183} (**J**), in which E176 was modified for SDASO-L, and P175 or E176 were modified for SDASO-M and SDASO-S (**K** and **L**).

Supplemental Figure 4. MS^n analyses of representative SDASO -L, -M and -S intra-linked peptides of BSA. MS^2 spectra of the intra-linked α_{intra} peptides (**A**) SDASO-L: m/z 474.8992^{3+}, (**B**) SDASO-M: m/z 455.8924^{3+}, (**C**) SDASO-S: m/z 451.2183^{3+}. MS^3 spectra of: (**D**) SDASO-L MS^2 fragment ion α_{A+T} (m/z 468.89^{3+}), (**E**) SDASO-M MS^2 fragment ion α_{A+T} (m/z 449.89^{3+}), (**F**) SDASO-S MS^2 fragment ion α_{A+T} (m/z 445.22^{3+}). The intra-linked peptides were determined as ^{25}DTHKSEIAHR^{34} with D25 linked to K28 for all three linkers.

Supplemental Figure 5. The SDASO XL-MS analysis workflow. SDASO cross-linking involves two steps: 1) lysine labeling by NHS ester; 2) photoactivated diazirine cross-linking of any AAs upon UV irradiation. path I-SDASO-L, path II-SDASO-M, and path III-SDASO-S.

Supplemental Figure 6. Reproducibility of SDASO XL-MS data for BSA. Comparisons of SDASO cross-linked peptide sequences among the three biological replicates for (**A**) SDASO-L, (**B**) SDASO-M, and (**C**) SDASO-S. Comparisons of SDASO K-X linkages among the three biological replicates for (**D**) SDASO-L, (**E**) SDASO-M, and (**F**) SDASO-S.

Supplemental Figure 7. Circular 2-D XL-maps of BSA. The maps were generated based on cross-links of BSA using (**A**) DSSO (4), (**B**) DHSO (5), (**C**) BMSO (6) and (**D**) the three SDASO linkers in this work.

Supplemental Figure 8. Structure-based quality control for the BSA dataset. Comparison of distance distributions of unique cross-links for (A) SDASO-S, (B) SDASO-M, and (C) SDASO-L with random cross-link distribution of BSA. The random distribution shown in grey was

generated by considering all possible pairwise distances between any K and any residue X (PDB: 4F5S).

Supplemental Figure 9. Reproducibility of SDASO XL-MS data for the 26S proteasome. Comparisons of SDASO cross-linked peptide sequences among the three biological replicates for (**A**) SDASO-L, (**B**) SDASO-M, and (**C**) SDASO-S. Comparisons of SDASO K-X linkages among the three biological replicates for (**D**) SDASO-L, (**E**) SDASO-M, and (**F**) SDASO-S. Note: the XL-MS data were generated from tryptic digests only.

Supplemental Figure 10. Comparison of SDASO XL-MS data for the 26S proteasome. Overlaps of the identified cross-linked peptide sequences using SDASO-L, SDASO-M, and SDASO-S respectively. Note: the XL-MS data were generated from tryptic digests only.

Supplemental Figure 11. Reproducibility of SDASO-L XL-MS data from chymotryptic digests of the 26S proteasome and comparison of data from trypsin and chymotrypsin digests of the 26S proteasome. Comparisons of SDASO-L (**A**) cross-linked peptide sequences and (**B**) K-X linkages among the three biological replicates of chymotryptic digests. Overlaps of the identified SDASO-L (**C**) cross-linked peptide sequences and (**D**) K-X linkages using trypsin and chymotrypsin digestions respectively. SDASO-L: tryptic digests; SDASO-Lc: chymotryptic digests.

Supplemental Figure 12. Distance distribution plots of SDASO cross-links mapped to the four known states of the yeast 26S proteasome structures (s1-s4). Respective cross-link distance distribution plots for SDASO-L, -M and -S cross-link data mapped onto (**A**) s1 (PDB: 4CR2), (**B**) s2 (PDB:4CR3), (**C**) s3 (PDB:4CR4) and (**D**) s4 (PDB:5MPC). Distance satisfaction thresholds are ≤30Å for SDASO-M and –S, and ≤35Å for SDASO-L.

Supplemental Figure 13. Structure-based quality control for the 26S proteasome SDASO XL-MS dataset. Comparison of distance distributions of unique cross-links for (A) SDASO-S, (B) SDASO-M, and (C) SDASO-L with random cross-link distribution of the yeast 26S proteasome. The random distribution shown in grey was generated by considering all possible pairwise distances between any K and any residue X (PDB: 4CR2).

Supplemental Figure 14. Distance distribution plots of the inter-subunit and intra-subunit K-X linkages mapped to the yeast 26S proteasome structures (s1-s4). (**A**) Average cross-link distance satisfaction rates across the four state models (s1: 4CR2, s2:4CR3, s3:4CR4, and s4:5MPD) for SDASO -L, -M, and –S, respectively. Respective distance distribution plots of SDASO inter-subunit and intra-subunit cross-links mapped onto (**B-D**) state s1 (PDB: 4CR2), (**E-G**) state s2 (PDB: 4CR3), (**H-J**) state s3 (PDB: 4CR4), and (**K-M**) state s4 (PDB: 5MPC) based on SDASO-L (**B, E, H, K**), SDASO-M (**C, F, I, L**) and SDASO-S (**D, G, J, M**) XL-MS data. Distance satisfaction thresholds are ≤30Å for SDASO-M and –S, and ≤35Å for SDASO-L.

Supplemental Figure 15. Distance distribution plots of the 19S RP and 20S CP K-X linkages mapped to the yeast 26S proteasome structures (s1-s4). (**A**) Average distance satisfaction rates across the four state models (s1: 4CR2, s2:4CR3, s3:4CR4, and s4:5MPD) for SDASO -L, -M, and -S K-X linkages of the 19S RP and 20S CP, respectively. Respective distance distribution plots of the 19S and 20S cross-links mapped onto (**B-D**) state s1 (PDB: 4CR2), (**E-G**) state s2 (PDB: 4CR3), (**H-J**) state s3 (PDB: 4CR4), and (**K-M**) state s4 (PDB: 5MPC) based on SDASO-L (**B, E, H, K**), SDASO-M (**C, F, I, L**) and SDASO-S (**D, G, J, M**) XL-MS data. Distance satisfaction thresholds are ≤30Å for SDASO-M and –S, and ≤35Å for SDASO-L.

Supplemental Figure 16. Reproducibility of DSSO XL-MS data for the 26S proteasome. Comparisons of DSSO (**A**) cross-linked peptide sequences and (**B**) K-K linkages among the two biological replicates of tryptic digests.

Supplemental Figure 17. Distance distribution analysis of DSSO cross-links of the 26S proteasome. (**A**) Average distance satisfaction rates of DSSO cross-links across the four state models (s1: 4CR2, s2:4CR3, s3:4CR4, and s4:5MPD) (distance satisfaction threshold for DSSO cross-links ≤ 30Å). (**B**) Respective distance distribution plots of DSSO cross-links mapped to the four states of the 26S proteasome structures. (**C**) Distribution of state-specific K-K linkages among the 14 possible state combinations. (**D**) Average distance satisfaction rates of inter-subunit and intra-subunit K-K linkages across the four state models. (**E**) Average distance satisfaction rates of the 19S and 20S K-K linkages across the four state models. (**F-I**) Respective distance distribution plots of inter-subunit and intra-subunit DSSO cross-links mapped to the four state models: (**F**) s1 (PDB: 4CR2), (**G**) s2 (PDB: 4CR3), (**H**) s3 (PDB: 4CR4) and (**I**) s4 (PDB: 5MPC). (**J-M**) Respective distance distribution plots of the 19S and 20S cross-links mapped to the four state models: (**J**) s1 (PDB: 4CR2), (**K**) s2 (PDB: 4CR3), (**L**) s3 (PDB: 4CR4) and (**M**) s4 (PDB: 5MPC).

Supplemental Figure 18. 3-D XL-Maps of SDASO and DSSO intra-subunit linkages of the 26S proteasome. (**A**) SDASO and (**B**) DSSO intra-subunit linkages (shown in yellow) mapped to s1 (PDB:4CR2).

Supplemental Figure 19. XL-maps of the Ecm29- and Ubp6-26S interactions. (**A**) 2-D XL-map describing the interactions of Ecm29 with Rpn1, Rpn6, Rpt1, and Rpt3-6. (**B**) 2-D XL-map illustrating the interactions of Ubp6 with Rpn1, Rpn2, Rpn8, Rpt1 and Rpt2. (Note: DSSO inter-subunit linkages are shown in blue and SDASO inter-subunit linkages are shown in green.)

Supplemental Figure 20. Variance in SDASO cross-linked sites. Distributions of the number of possible cross-linked site locations in the identified SDASO cross-links due to ambiguity in site identification for (**A**) BSA and (**B**) 26S Proteasome.

Supplemental Figure 21. Respective distribution of amino acids targeted by diazirine based on the identified SDASO cross-links in (**A**) 26S proteasome and (B) BSA for SDASO -L, -M, and-S linkers.

References

1. Liang, J., Zhang, L., Tan, X. L., Qi, Y. K., Feng, S., Deng, H., Yan, Y., Zheng, J. S., Liu, L., and Tian, C. L. (2017) Chemical Synthesis of Diubiquitin-Based Photoaffinity Probes for Selectively Profiling Ubiquitin-Binding Proteins. *Angew Chem Int Ed Engl* 56, 2744-2748
2. Sun, R., Yin, L., Zhang, S., He, L., Cheng, X., Wang, A., Xia, H., and Shi, H. (2017) Simple Light-Triggered Fluorescent Labeling of Silica Nanoparticles for Cellular Imaging Applications. *Chemistry* 23, 13893-13896
3. Ma, L., Tu, C., Le, P., Chitoor, S., Lim, S. J., Zahid, M. U., Teng, K. W., Ge, P., Selvin, P. R., and Smith, A. M. (2016) Multidentate Polymer Coatings for Compact and Homogeneous Quantum Dots with Efficient Bioconjugation. *Journal of the American Chemical Society* 138, 3382-3394
4. Kao, A., Chiu, C. L., Vellucci, D., Yang, Y., Patel, V. R., Guan, S., Randall, A., Baldi, P., Rychnovsky, S. D., and Huang, L. (2011) Development of a novel cross-linking strategy for fast and accurate identification of cross-linked peptides of protein complexes. *Molecular & cellular proteomics : MCP* 10, M110.002212
5. Gutierrez, C. B., Yu, C., Novitsky, E. J., Huszagh, A. S., Rychnovsky, S. D., and Huang, L. (2016) Developing an Acidic Residue Reactive and Sulfoxide-Containing MS-Cleavable Homobifunctional Cross-Linker for Probing Protein-Protein Interactions. *Analytical chemistry*
6. Gutierrez, C. B., Block, S. A., Yu, C., Soohoo, S. M., Huszagh, A. S., Rychnovsky, S. D., and Huang, L. (2018) Development of a Novel Sulfoxide-Containing MS-Cleavable Homobifunctional Cysteine-Reactive Cross-Linker for Studying Protein-Protein Interactions. *Analytical chemistry* 90, 7600-7607

Annotated spectra for cross-link identifications can be viewed through MS-Viewer (https://msviewer.ucsf.edu/prospector/cgi-bin/msform.cgi?form=msviewer) using the provided links:

1. **Spectra for cross-linked peptides of BSA;**

   SDASO-S (https://msviewer.ucsf.edu:443/prospector/cgi-bin/mssearch.cgi?report_title=MS-Viewer&search_key=x65tqdukzv&search_name=msviewer)

   SDASO-M (https://msviewer.ucsf.edu:443/prospector/cgi-bin/mssearch.cgi?report_title=MS-Viewer&search_key=8jj2ya8grp&search_name=msviewer)

   SDASO-L (https://msviewer.ucsf.edu:443/prospector/cgi-bin/mssearch.cgi?report_title=MS-Viewer&search_key=efsepaedxv&search_name=msviewer)

2. **Spectra for cross-linked peptides of 26S proteasome;**

   SDASO-S (https://msviewer.ucsf.edu:443/prospector/cgi-bin/mssearch.cgi?report_title=MS-Viewer&search_key=ood8apmfhr&search_name=msviewer)

   SDASO-M (https://msviewer.ucsf.edu:443/prospector/cgi-bin/mssearch.cgi?report_title=MS-Viewer&search_key=lud3axrrfm&search_name=msviewer)

   SDASO-L (https://msviewer.ucsf.edu:443/prospector/cgi-bin/mssearch.cgi?report_title=MS-Viewer&search_key=1cegjbxqaf&search_name=msviewer)

   DSSO (https://msviewer.ucsf.edu:443/prospector/cgi-bin/mssearch.cgi?report_title=MS-Viewer&search_key=bbpm76tsng&search_name=msviewer)

Figure S-1

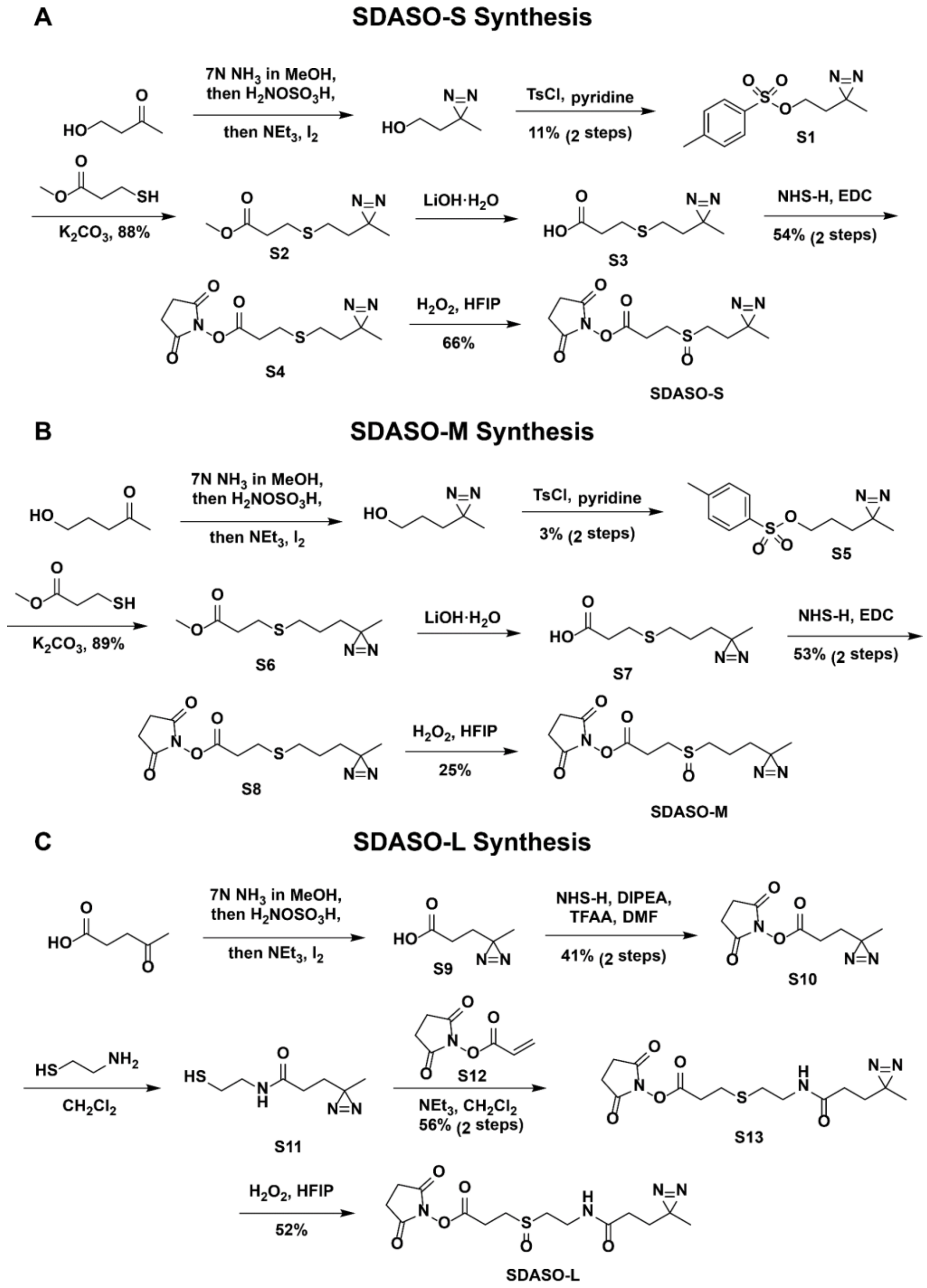
A
SDASO-S Synthesis
7N NH3 in MeOH, then H2NOSO3H,
then NEt3, I2
TsCl, pyridine
11% (2 steps)
S1
K2CO3, 88%
S2
LiOH·H2O
S3
NHS-H, EDC
54% (2 steps)
S4
H2O2, HFIP
66%
SDASO-S
B
SDASO-M Synthesis
7N NH3 in MeOH, then H2NOSO3H,
then NEt3, I2
TsCl, pyridine
3% (2 steps)
S5
K2CO3, 89%
S6
LiOH·H2O
S7
NHS-H, EDC
53% (2 steps)
S8
H2O2, HFIP
25%
SDASO-M
C
SDASO-L Synthesis
7N NH3 in MeOH, then H2NOSO3H,
then NEt3, I2
S9
NHS-H, DIPEA, TFAA, DMF
41% (2 steps)
S10
CH2Cl2
S11
S12
NEt3, CH2Cl2
56% (2 steps)
S13
H2O2, HFIP
52%
SDASO-L

Figure S-2

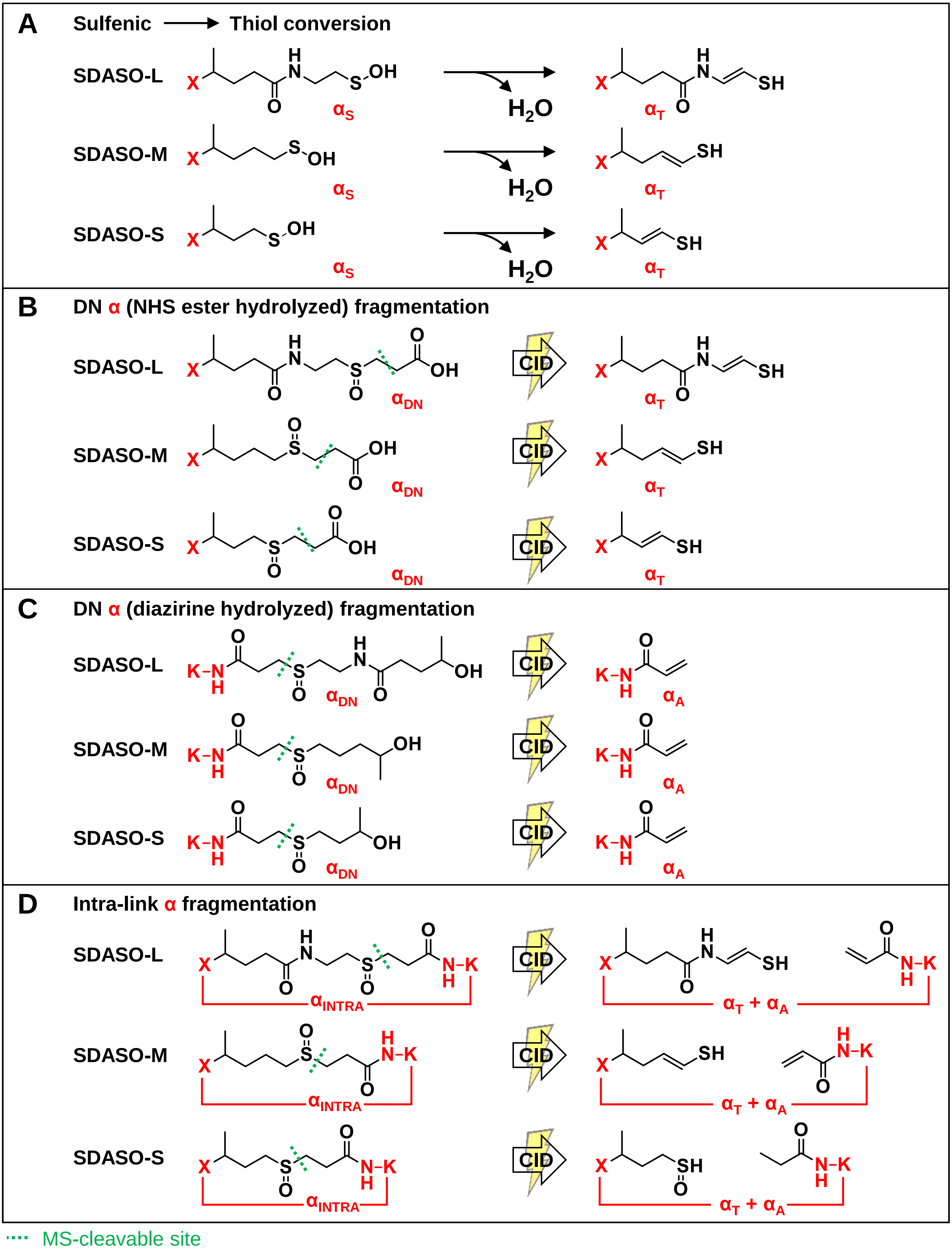

A
Sulfenic → Thiol conversion
SDASO-L
SDASO-M
SDASO-S
αS
αT
H2O
B
DN α (NHS ester hydrolyzed) fragmentation
SDASO-L
SDASO-M
SDASO-S
αDN
αT
CID
C
DN α (diazirine hydrolyzed) fragmentation
SDASO-L
SDASO-M
SDASO-S
αDN
αA
CID
D
Intra-link α fragmentation
SDASO-L
SDASO-M
SDASO-S
αINTRA
αT + αA
CID
MS-cleavable site

Figure S-3

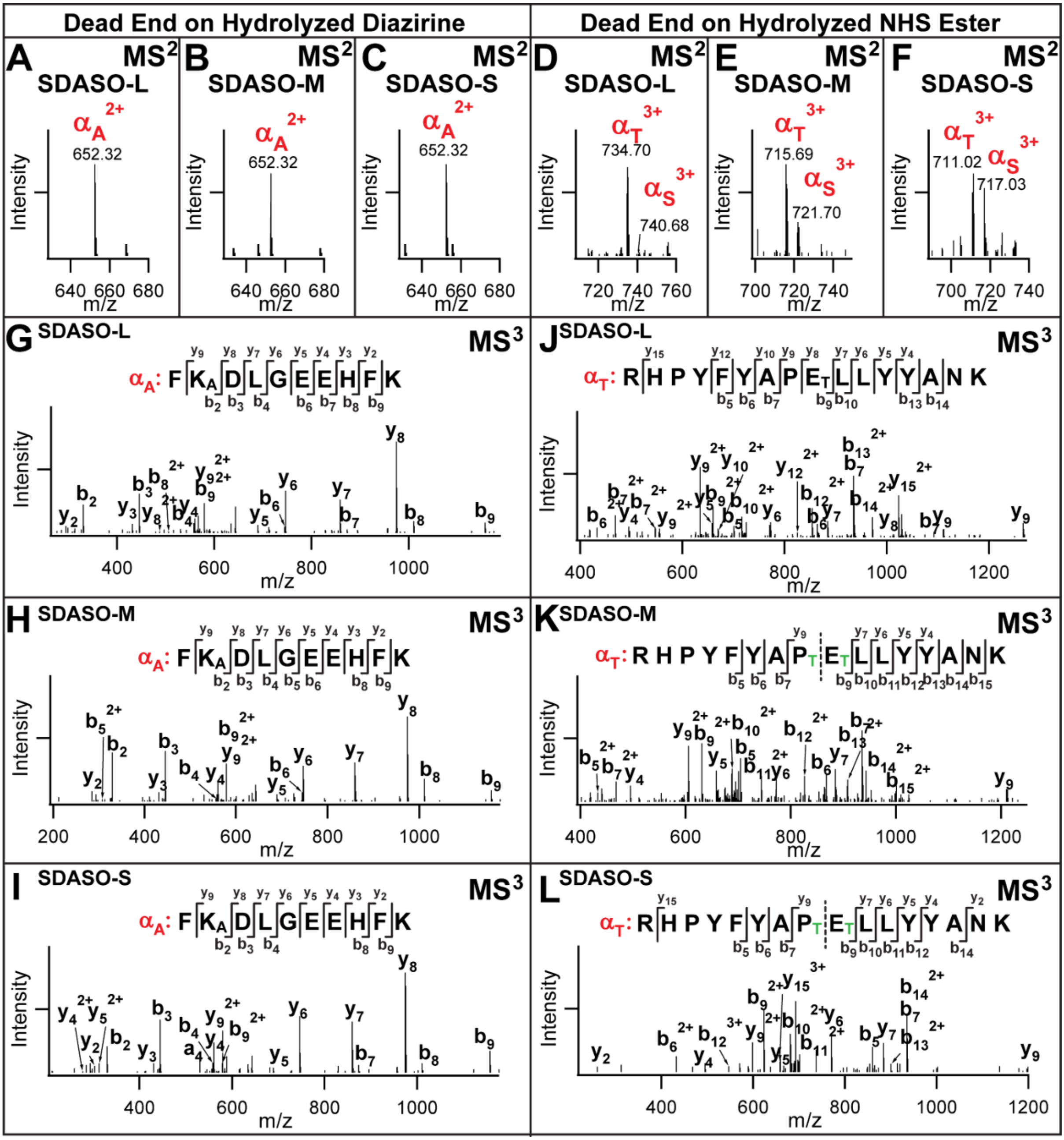

Dead End on Hydrolyzed Diazirine
Dead End on Hydrolyzed NHS Ester
A MS2 SDASO-L
B MS2 SDASO-M
C MS2 SDASO-S
D MS2 SDASO-L
E MS2 SDASO-M
F MS2 SDASO-S
G SDASO-L MS3
H SDASO-M MS3
I SDASO-S MS3
J SDASO-L MS3
K SDASO-M MS3
L SDASO-S MS3
αA: FKADLGEEHFK
αT: RHPYFYAPETLLYYANK
Intensity
m/z

Figure S-4

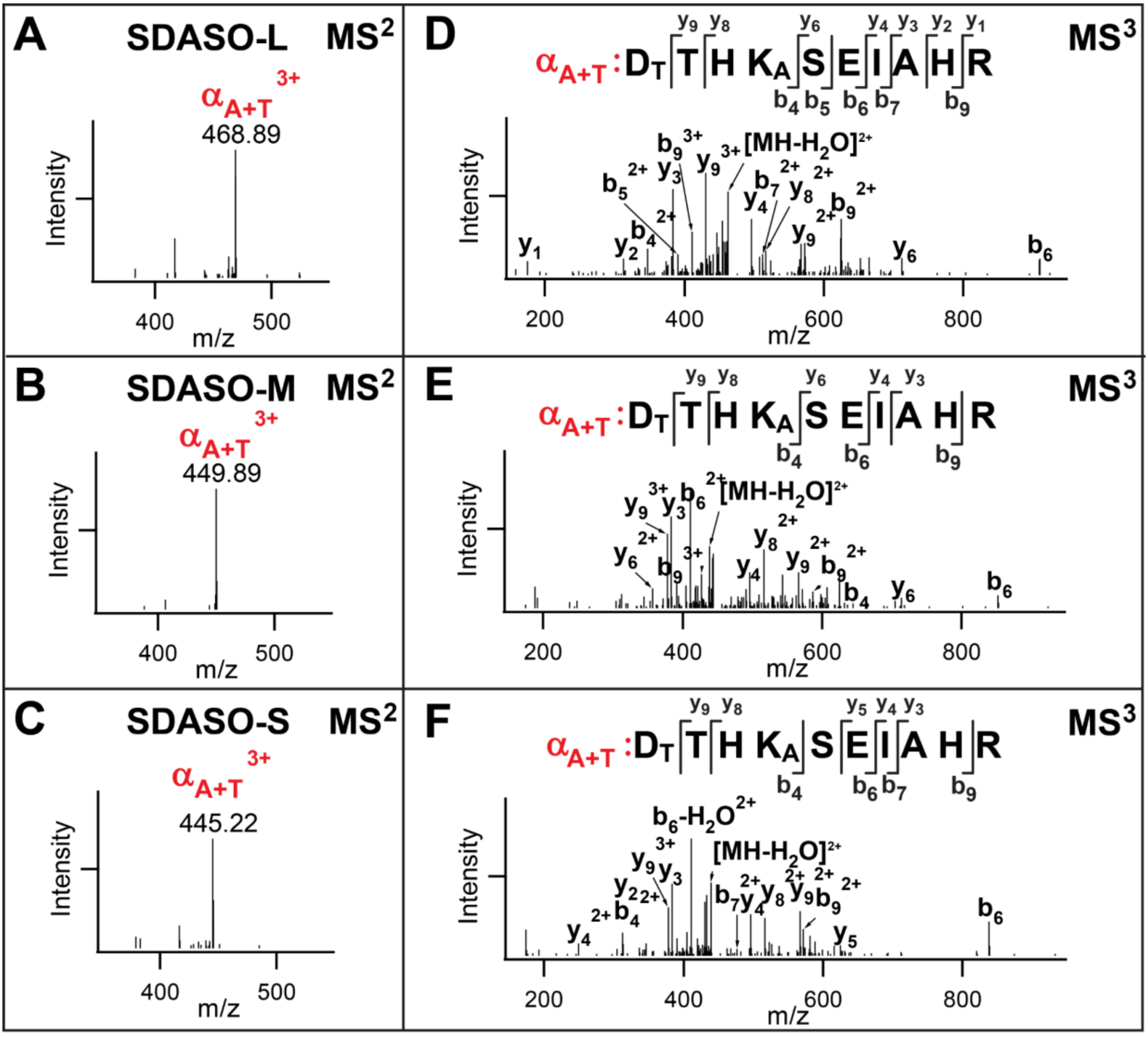

A
SDASO-L
MS2
B
SDASO-M
MS2
C
SDASO-S
MS2
D
MS3
E
MS3
F
MS3
468.89
449.89
445.22
Intensity
m/z
[MH-H2O]2+

Figure S-5

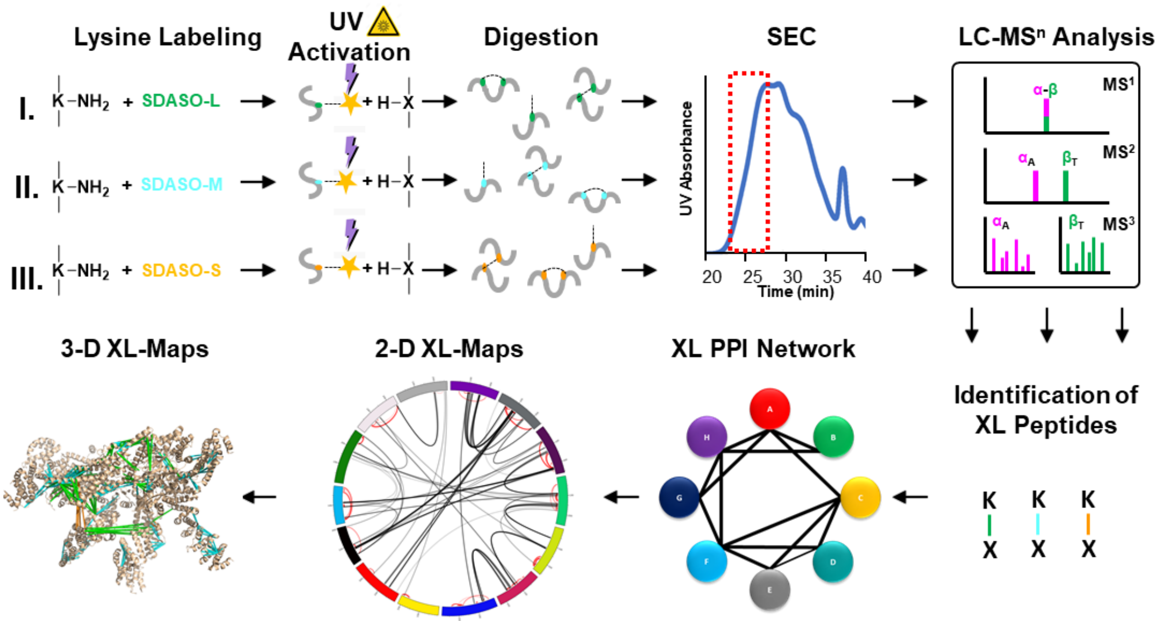

Lysine Labeling
UV
Activation
Digestion
SEC
LC-MSⁿ Analysis
I.
II.
III.
K–NH2 + SDASO-L
K–NH2 + SDASO-M
K–NH2 + SDASO-S
+ H–X
UV Absorbance
20 25 30 35 40
Time (min)
MS1
MS2
MS3
α-β
αA
βT
3-D XL-Maps
2-D XL-Maps
XL PPI Network
Identification of
XL Peptides
K
X
A
B
C
D
E
F
G
H

Figure S-6

BSA XL-Peptide Triplicate Reproducibility

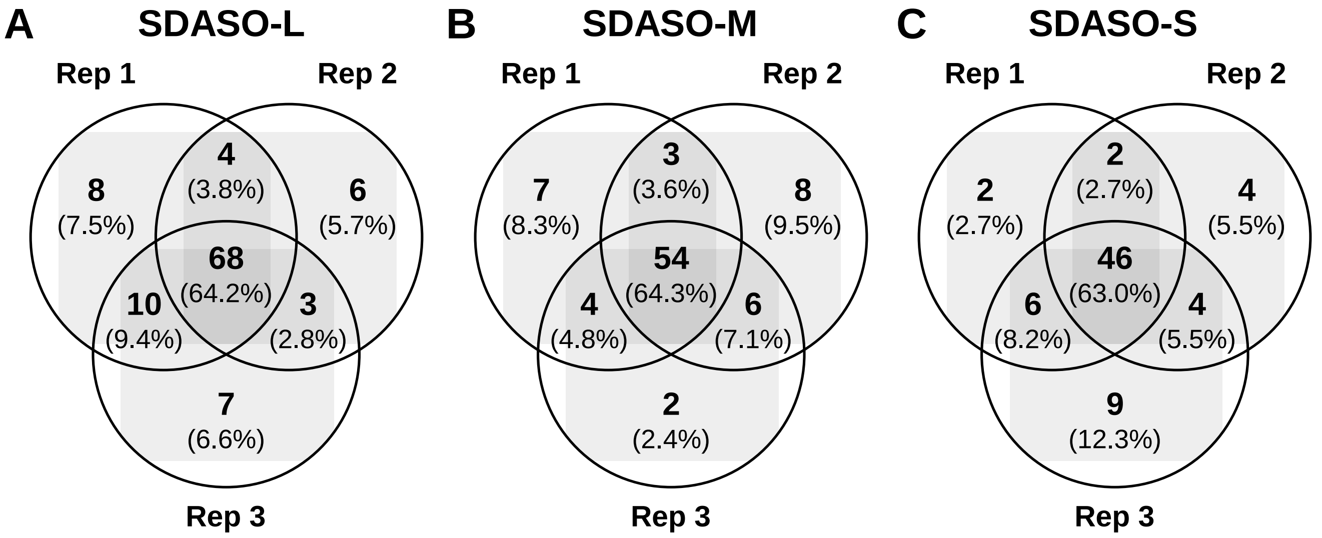

BSA K-X Linkage Triplicate Reproducibility

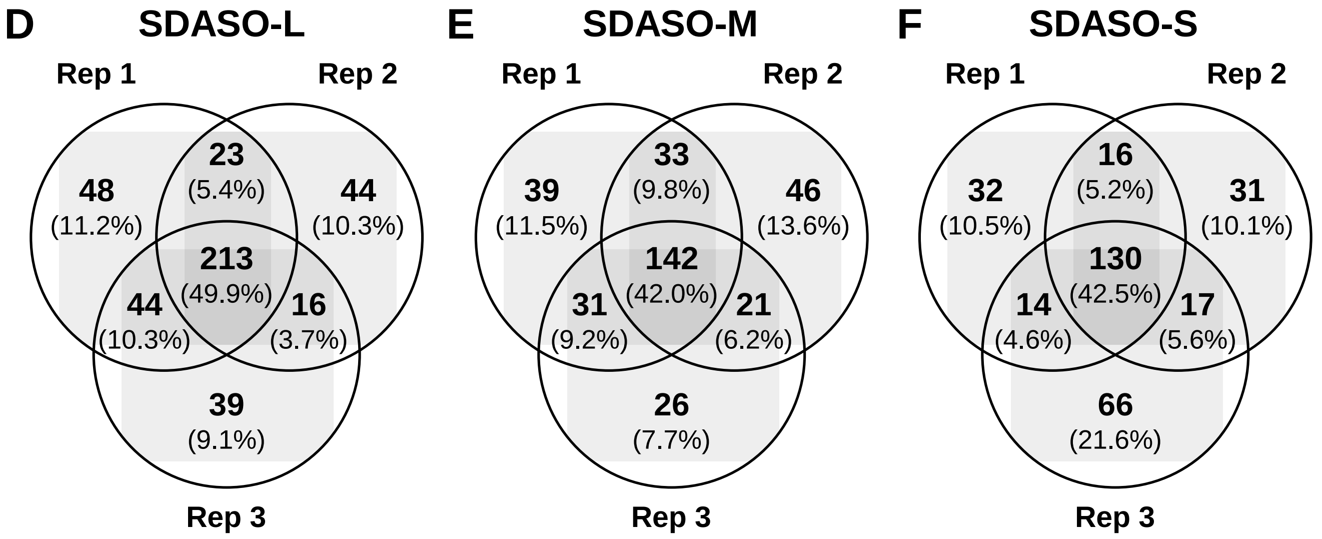

Figure S-7

BSA 2-D XL-Maps

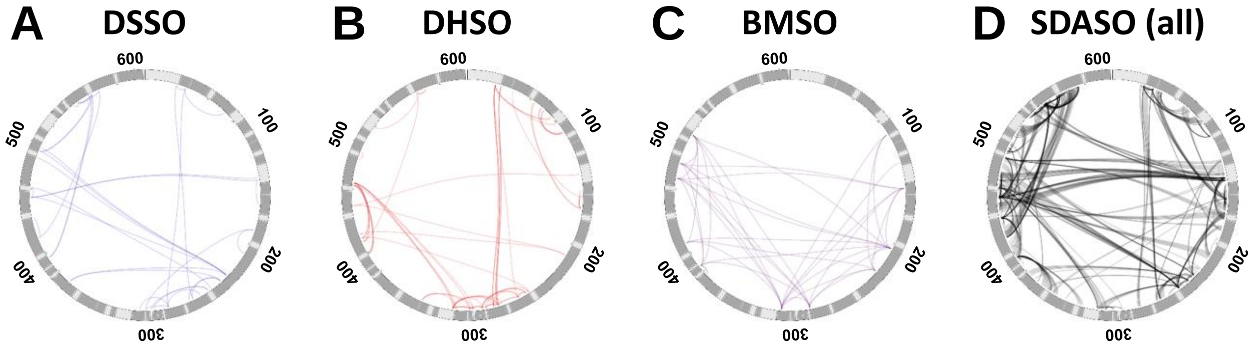

Figure S-8

A

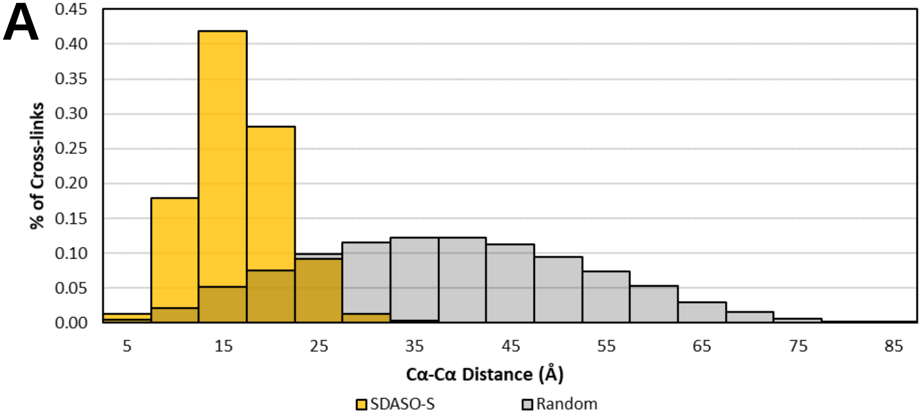

B

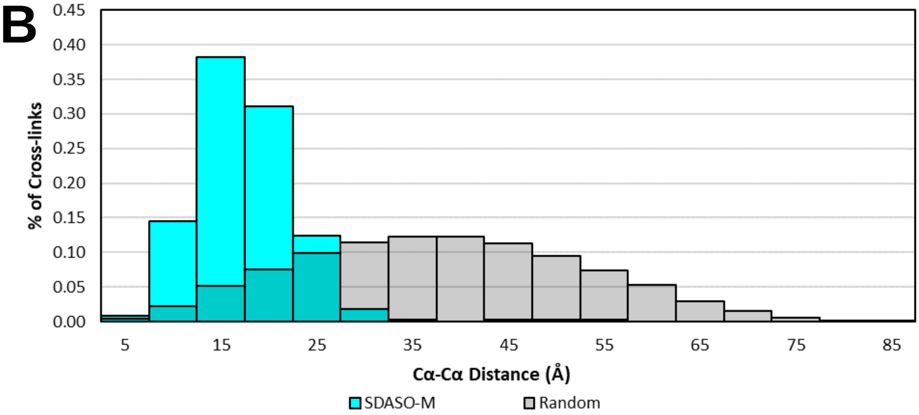

C

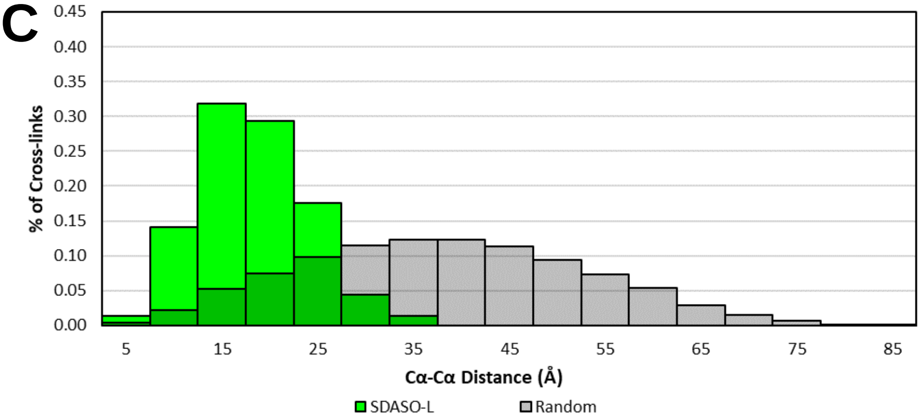

Figure S-9

26S XL-Peptide Triplicate Reproducibility

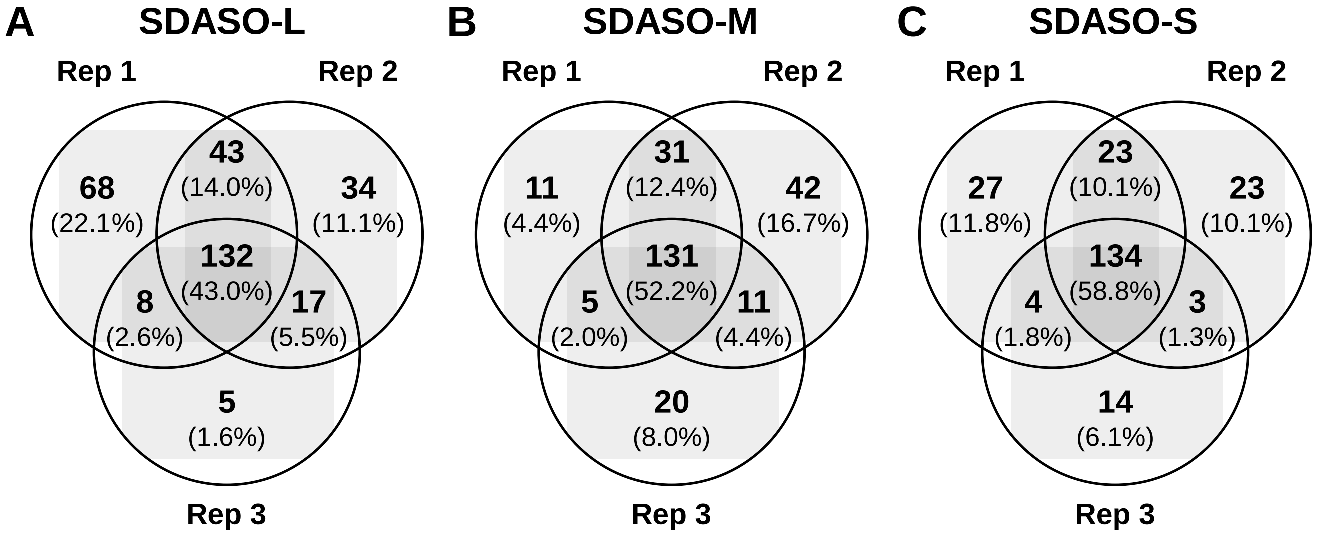

K-X Linkage Triplicate Reproducibility

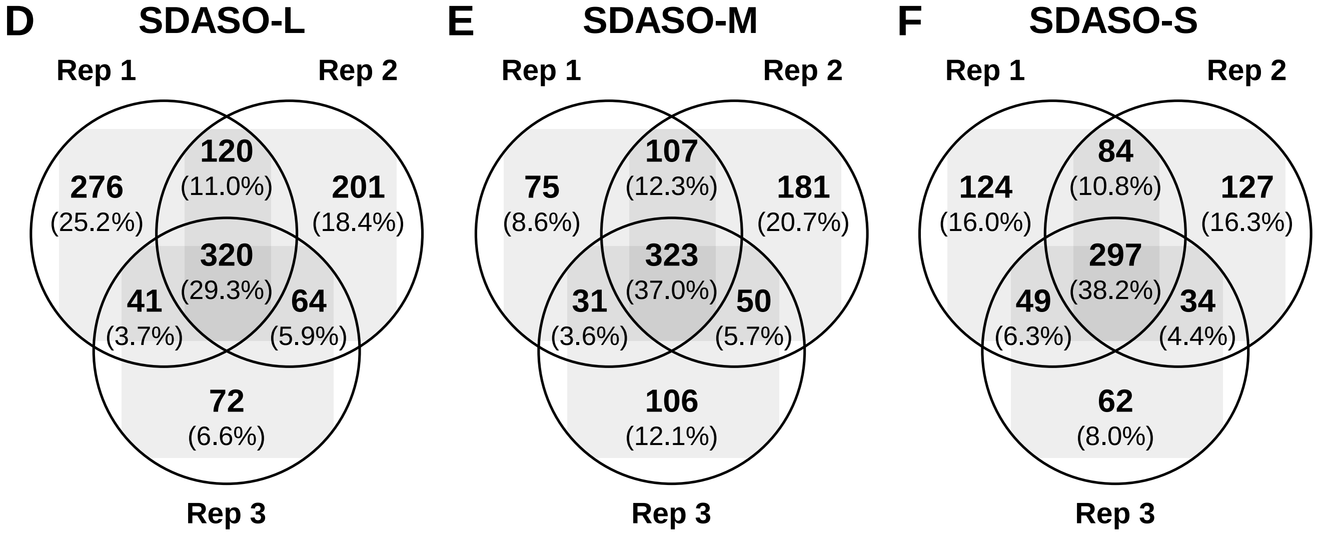

Figure S-10

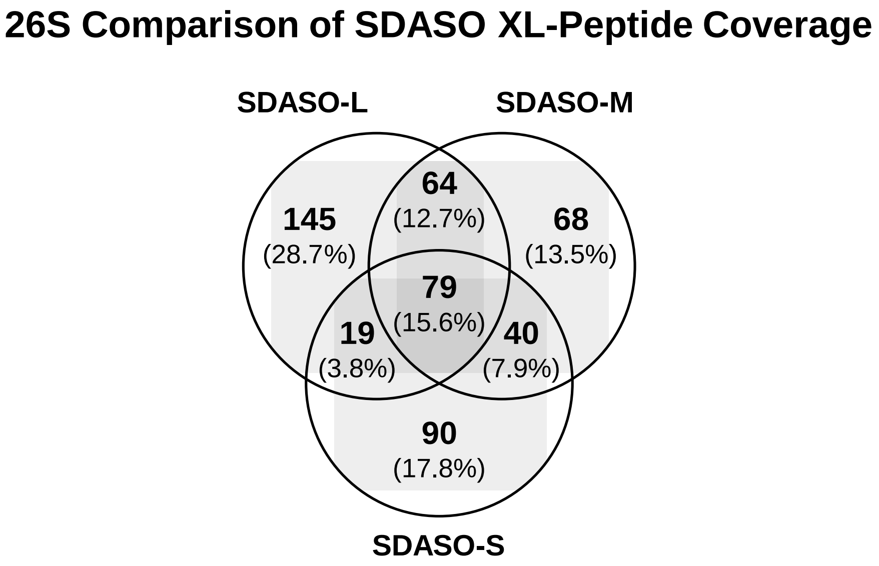

26S Comparison of SDASO XL-Peptide Coverage
SDASO-L
SDASO-M
145
(28.7%)
64
(12.7%)
68
(13.5%)
79
(15.6%)
19
(3.8%)
40
(7.9%)
90
(17.8%)
SDASO-S

Figure S-11

Reproducibility of Chymotrypsin Digested SDASO-L XL 26S

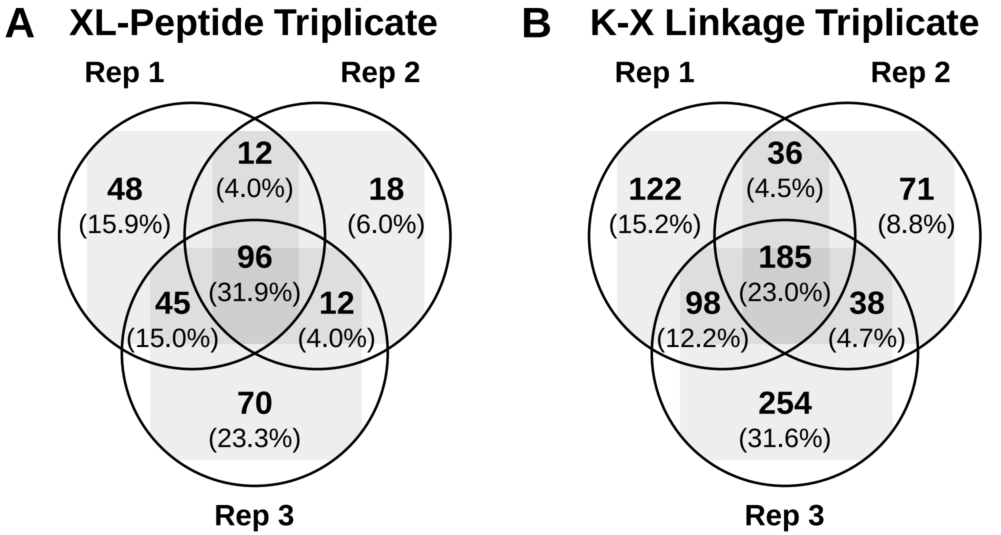

Comparison of Chymotrypsin vs. Trypsin Digested SDASO-L XL 26S

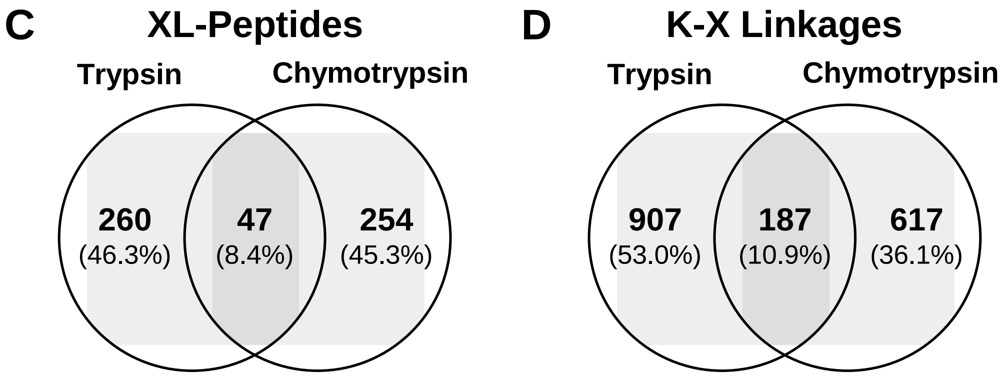

Figure S-12

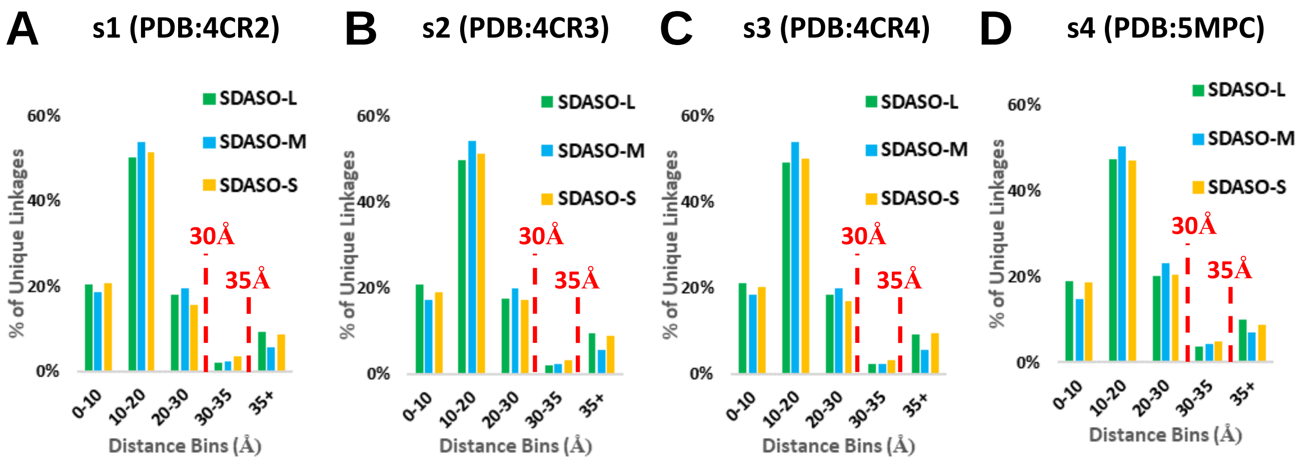

A
s1 (PDB:4CR2)
B
s2 (PDB:4CR3)
C
s3 (PDB:4CR4)
D
s4 (PDB:5MPC)
SDASO-L
SDASO-M
SDASO-S
% of Unique Linkages
60%
40%
20%
0%
30Å
35Å
0-10
10-20
20-30
30-35
35+
Distance Bins (Å)

Figure S-13

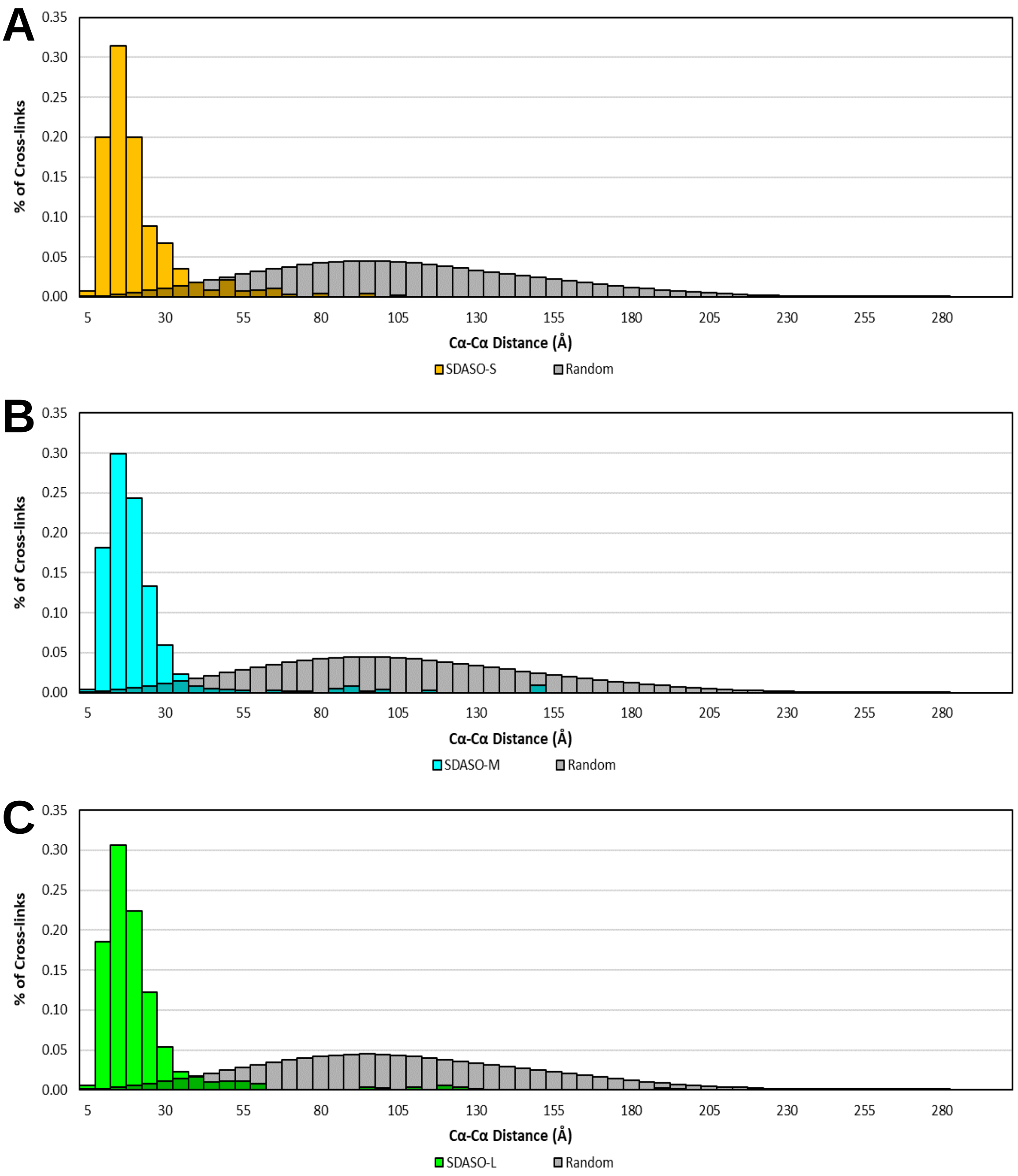
A
0.35
0.30
0.25
0.20
0.15
0.10
0.05
0.00
% of Cross-links
5
30
55
80
105
130
155
180
205
230
255
280
Cα-Cα Distance (Å)
SDASO-S
Random
B
% of Cross-links
Cα-Cα Distance (Å)
SDASO-M
Random
C
% of Cross-links
Cα-Cα Distance (Å)
SDASO-L
Random

Figure S-14

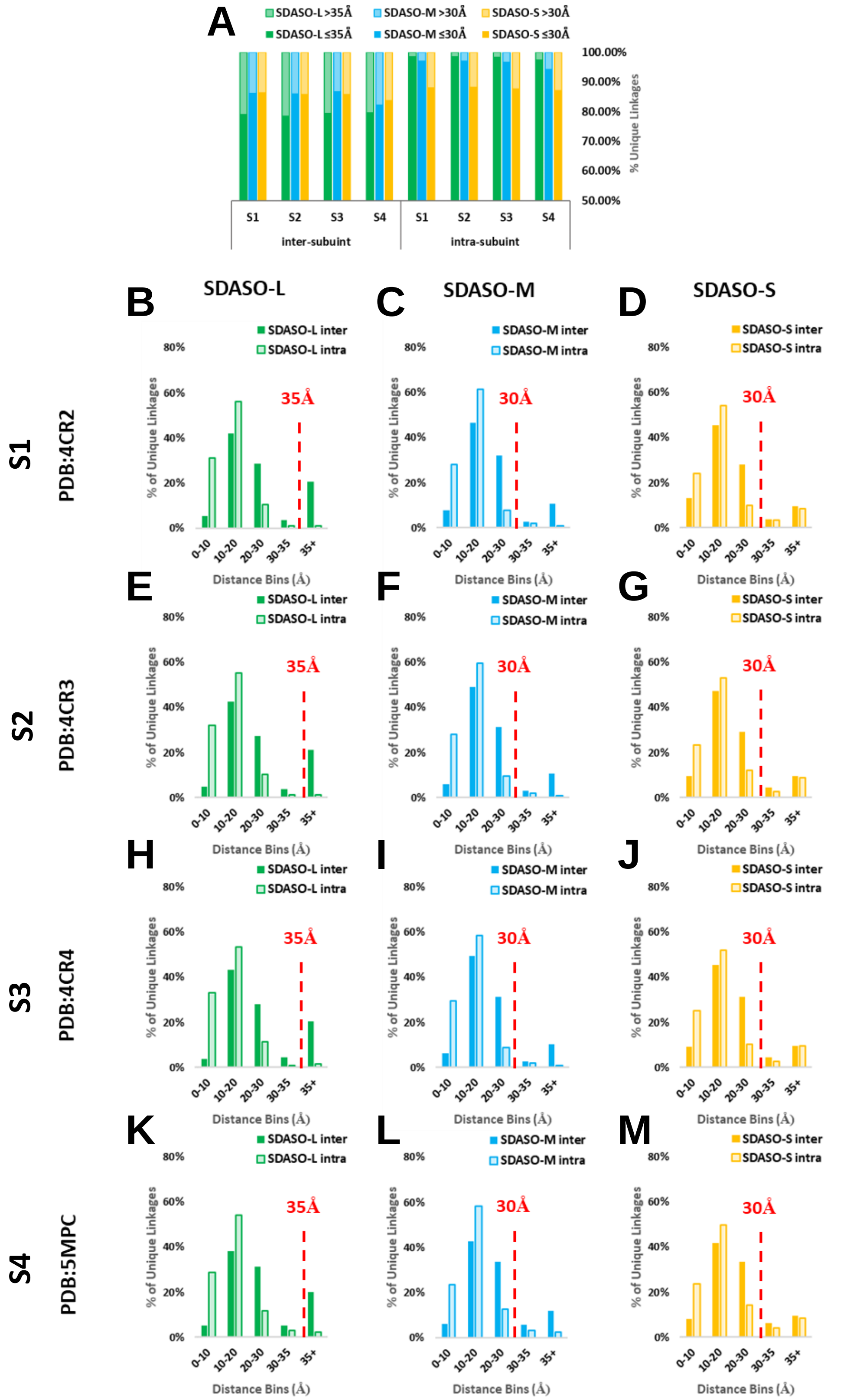

A
SDASO-L >35Å
SDASO-M >30Å
SDASO-S >30Å
SDASO-L ≤35Å
SDASO-M ≤30Å
SDASO-S ≤30Å
% Unique Linkages
inter-subuint
intra-subuint
B
C
D
SDASO-L
SDASO-M
SDASO-S
S1
PDB:4CR2
S2
PDB:4CR3
S3
PDB:4CR4
S4
PDB:5MPC
E
F
G
H
I
J
K
L
M
SDASO-L inter
SDASO-L intra
SDASO-M inter
SDASO-M intra
SDASO-S inter
SDASO-S intra
35Å
30Å
% of Unique Linkages
Distance Bins (Å)

Figure S-15

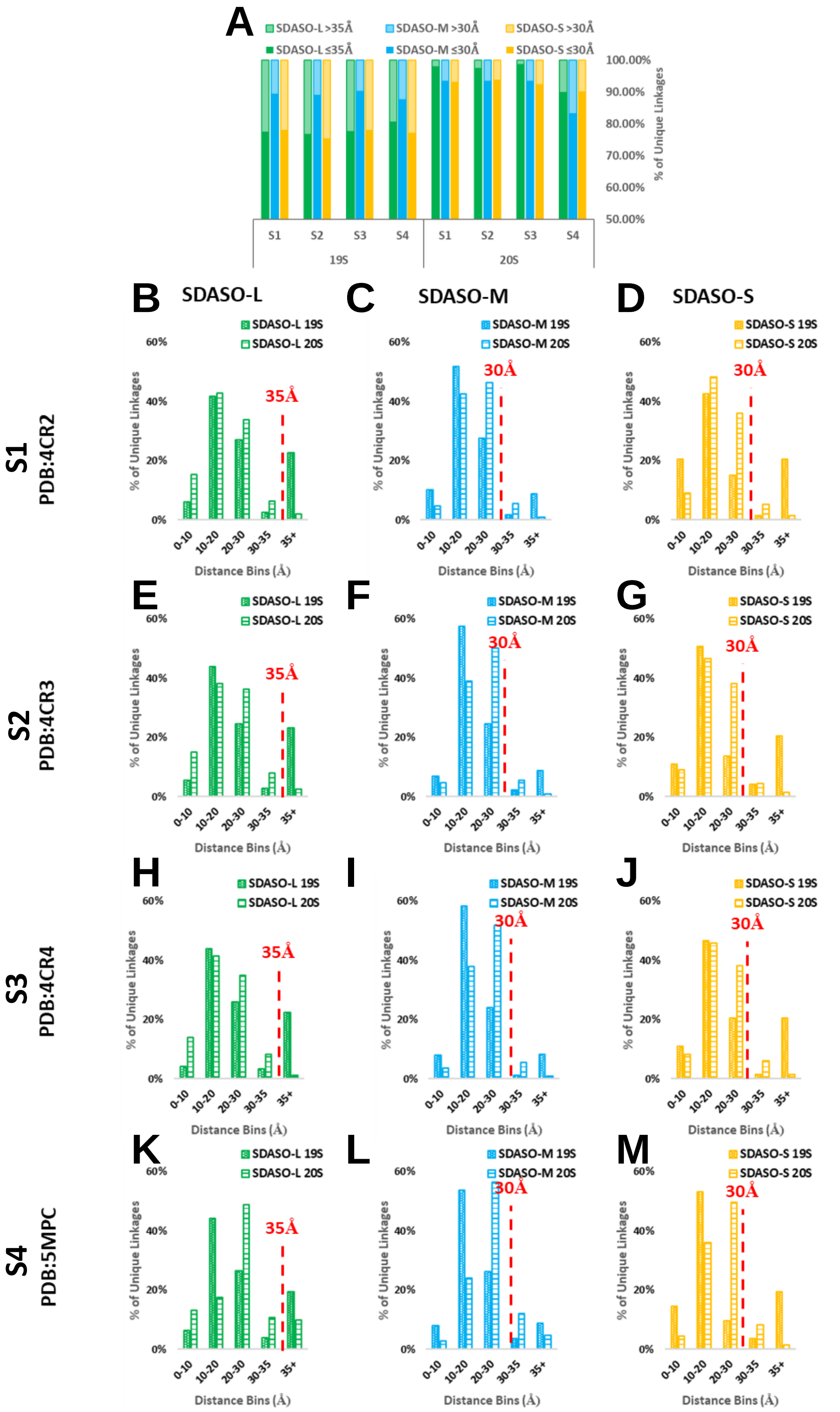
A
SDASO-L >35Å
SDASO-M >30Å
SDASO-S >30Å
SDASO-L ≤35Å
SDASO-M ≤30Å
SDASO-S ≤30Å
% of Unique Linkages
100.00%
90.00%
80.00%
70.00%
60.00%
50.00%
S1 S2 S3 S4 S1 S2 S3 S4
19S
20S
B SDASO-L
C SDASO-M
D SDASO-S
E
F
G
H
I
J
K
L
M
S1 PDB:4CR2
S2 PDB:4CR3
S3 PDB:4CR4
S4 PDB:5MPC
SDASO-L 19S
SDASO-L 20S
SDASO-M 19S
SDASO-M 20S
SDASO-S 19S
SDASO-S 20S
35Å
30Å
60%
40%
20%
0%
0-10
10-20
20-30
30-35
35+
Distance Bins (Å)

Figure S-16

26S DSSO XL Reproducibility

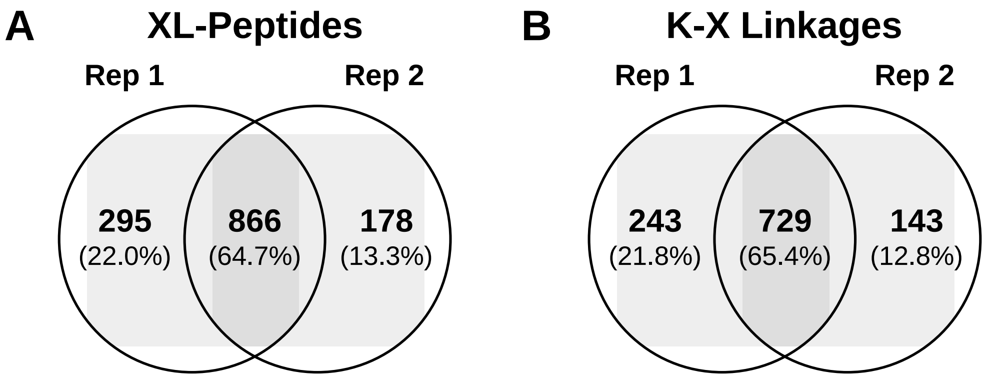

Figure S-17

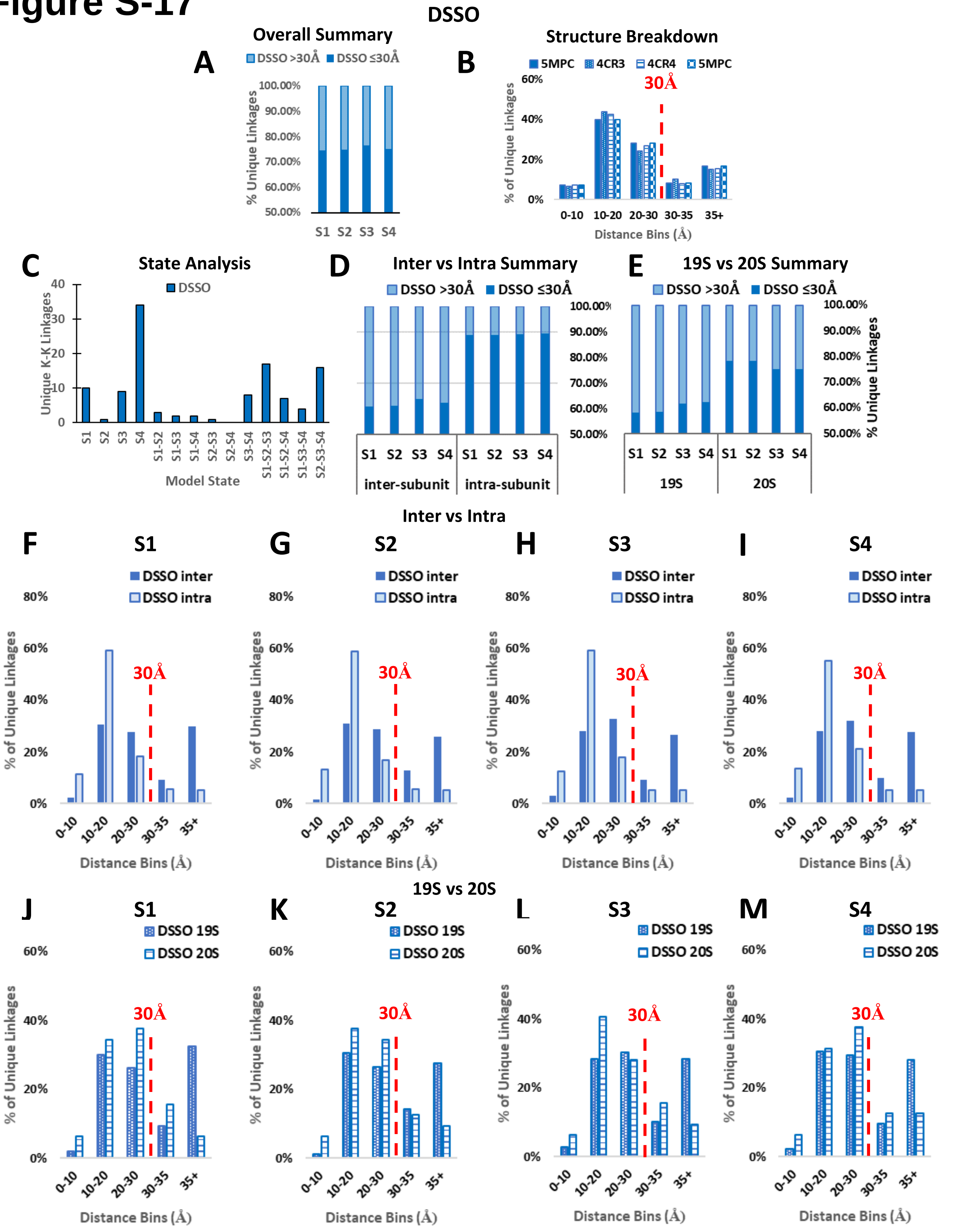

DSSO
A
Overall Summary
DSSO >30Å
DSSO ≤30Å
% Unique Linkages
100.00%
90.00%
80.00%
70.00%
60.00%
50.00%
S1
S2
S3
S4
B
Structure Breakdown
5MPC
4CR3
4CR4
5MPC
30Å
% of Unique Linkages
60%
40%
20%
0%
0-10
10-20
20-30
30-35
35+
Distance Bins (Å)
C
State Analysis
DSSO
Unique K-K Linkages
40
30
20
10
0
S1
S2
S3
S4
S1-S2
S1-S3
S1-S4
S2-S3
S2-S4
S3-S4
S1-S2-S3
S1-S2-S4
S1-S3-S4
S2-S3-S4
Model State
D
Inter vs Intra Summary
DSSO >30Å
DSSO ≤30Å
inter-subunit
intra-subunit
E
19S vs 20S Summary
DSSO >30Å
DSSO ≤30Å
% Unique Linkages
19S
20S
Inter vs Intra
F
S1
G
S2
H
S3
I
S4
DSSO inter
DSSO intra
80%
60%
40%
20%
0%
30Å
% of Unique Linkages
0-10
10-20
20-30
30-35
35+
Distance Bins (Å)
19S vs 20S
J
S1
K
S2
L
S3
M
S4
DSSO 19S
DSSO 20S
60%
40%
20%
0%
30Å
% of Unique Linkages
Distance Bins (Å)

Figure S-18

Intra-Subunit 3-D XL-Maps of the 26S

A **SDASO** **B** **DSSO**

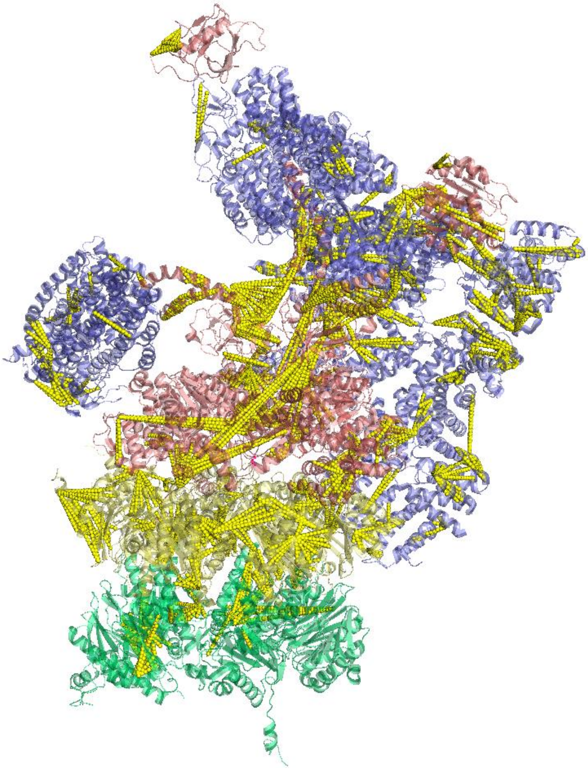

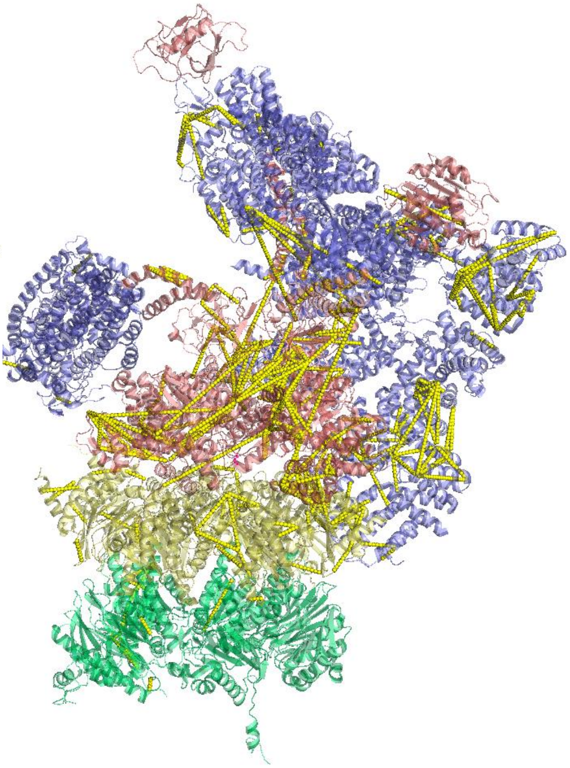

s1 (PDB:4CR2)

Figure S-19

A ECM29

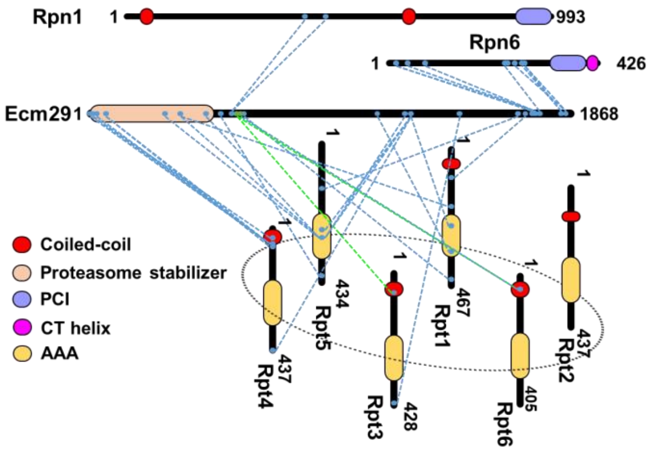

B Ubp6

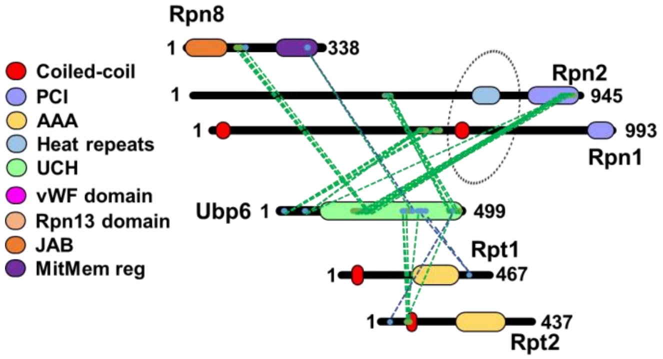

Figure S-20

Variance in XL-Sites

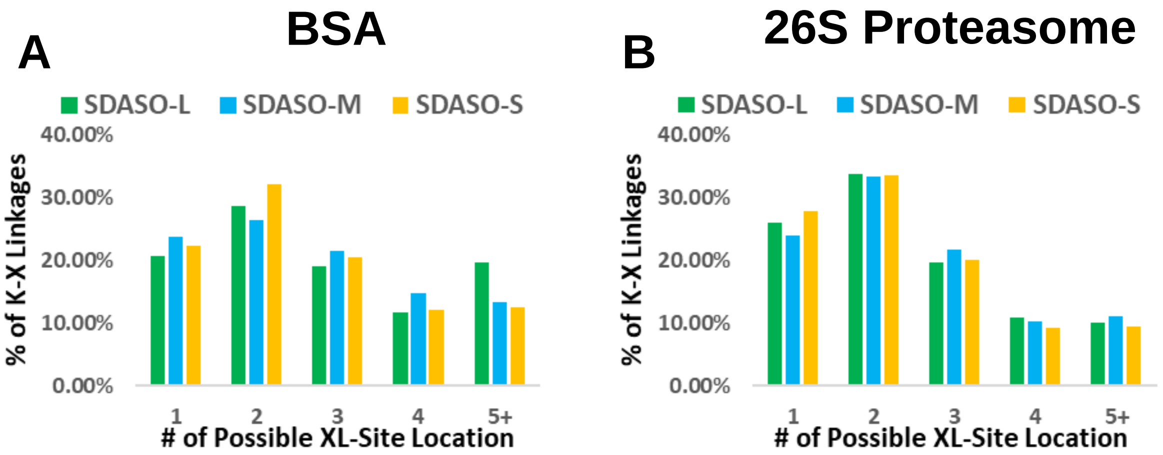

Figure S-21

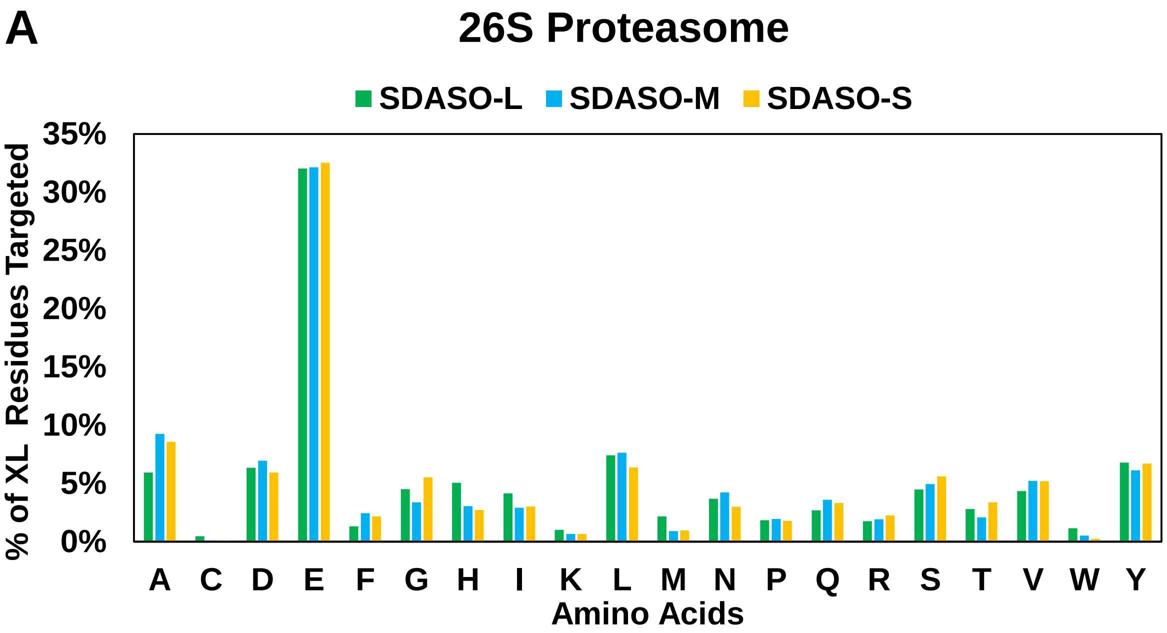
A
26S Proteasome
SDASO-L
SDASO-M
SDASO-S
% of XL Residues Targeted
35%
30%
25%
20%
15%
10%
5%
0%
A C D E F G H I K L M N P Q R S T V W Y
Amino Acids

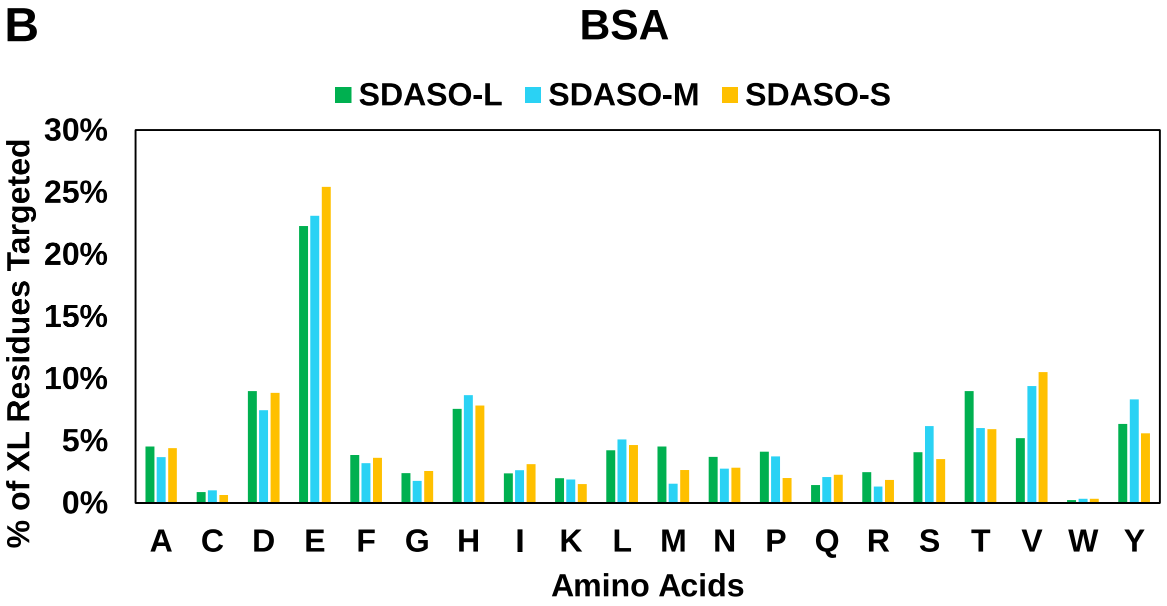
B
BSA
SDASO-L
SDASO-M
SDASO-S
% of XL Residues Targeted
30%
25%
20%
15%
10%
5%
0%
A C D E F G H I K L M N P Q R S T V W Y
Amino Acids
